# Toxicological and molecular profiling of insecticide resistance in a Brazilian strain of fall armyworm resistant to Bt Cry1 proteins

**DOI:** 10.1002/ps.6061

**Published:** 2020-09-10

**Authors:** Debora Boaventura, Benjamin Buer, Niklas Hamaekers, Frank Maiwald, Ralf Nauen

**Affiliations:** ^1^ Institute of Crop Science and Resource Conservation, University of Bonn Bonn Germany; ^2^ Bayer AG, Crop Science Division R&D Pest Control Monheim Germany

**Keywords:** fall armyworm, cross‐resistance, detoxification enzymes, resistance management

## Abstract

**Background:**

*Spodoptera frugiperda*, fall armyworm (FAW) is the major pest of maize in Brazil and has readily acquired field resistance to a broad range of synthetic insecticides and to *Bacillus thuringiensis* (Bt) insecticidal proteins expressed in important crops. This study aims to understand patterns of cross‐resistance in FAW by investigating the toxicological profile of a Bt‐resistant Brazilian strain (Sf_Des) in comparison to a Bt‐susceptible strain (Sf_Bra).

**Results:**

Laboratory bioassays with 15 active substances of nine mode of action classes revealed that Sf_Des has a medium level of resistance to deltamethrin and chlorpyrifos. Very high cross‐resistance was observed among Cry1 toxins, but high susceptibility against Vip3A. Strain Sf_Des exhibited – depending on the substrate – up to 19‐fold increased cytochrome P450 activity in comparison to Sf_Bra. RNA‐Seq data support a major role of P450 enzymes in the detoxification of insecticides because we detected 85 P450 transcripts upregulated in Sf_Des. Quantitative reverse transcription polymerase chain reaction (RT‐qPCR) analysis confirmed that *CYP9A*‐like and *CYP6B39* are significantly upregulated (>200‐fold) in Sf_Des in comparison to Sf_Bra strain. No target‐site mutation linked to pyrethroid resistance was detected, but mutations in the AChE linked to organophosphate resistance were observed in Sf_Des. A Gene Ontology (GO) analysis of differentially expressed genes (DEG) categorized most of them into the biological process category, involved in oxidation–reduction and metabolic processes.

**Conclusion:**

Our results indicate that multiple/cross‐resistance mechanisms may have developed in the Sf_Des strain to conventional insecticides and Bt insecticidal proteins. The systematic toxicological analysis presented will help to guide recommendations for an efficient resistance management. © 2020 The Authors. *Pest Management Science* published by John Wiley & Sons Ltd on behalf of Society of Chemical Industry.

## Graphical abstract

*Spodoptera frugiperda*, fall armyworm (FAW) is the major pest of maize in Brazil and has readily acquired field resistance to a broad range of synthetic insecticides and to *Bacillus thuringiensis* (Bt) insecticidal proteins expressed in important crops. This study aims to understand patterns of cross‐resistance in FAW by investigating the toxicological profile of a Bt‐resistant Brazilian strain (Sf_Des) in comparison to a Bt‐susceptible strain (Sf_Bra) at molecular and biochemical level.

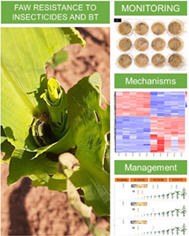

## 1 INTRODUCTION

The fall armyworm (FAW), *Spodoptera frugiperda* (J.E. Smith, 1797) (Lepidoptera: Noctuidae), is native to the American continent where it is the major pest of maize.^1^, ^2^ However, since 2016, FAW has rapidly invaded the tropical and subtropical regions of the Eastern hemisphere, becoming a pest of global economic relevance.^3^, ^4^, ^5^, ^6^ FAW control has relied intensively on chemical insecticides, prompting resistance to many classes of insecticides ^7^, ^8^ and currently, FAW is among the top 15 most resistant insect pest species worldwide.^9^ In Brazil, cases of insecticide resistance have been reported for different chemical classes including organophosphates, pyrethroids, spinosyns, benzoylureas and (lately) diamides.^10^, ^11^, ^12^, ^13^, ^14^

The commercialization of genetically engineered crops expressing insecticidal crystal (Cry) or/and vegetative (Vip) proteins derived from the bacterium *Bacillus thuringiensis* (Bt) Berliner has considerably reduced the number of insecticide applications for the control of lepidopteran pests, including FAW.^15^ In Brazil, the refuge (cultivation of non‐Bt nearby Bt crops) strategy is highly recommended to delay the onset of resistance to Bt crops ^16^ and the refuge area can be treated up to two times (including seed treatment) during the growing season with non‐Bt based foliar insecticide sprays until V6 stage.^17^, ^18^ Despite the high adoption of Bt crops in Brazil (51.3 million ha), there is rather low compliance with regard to the proposed refuge strategy.^19^ Furthermore, not all Bt proteins are high‐dose for FAW – thus, the respective protein expressed *in planta* does not cause 100% mortality of insects feeding on it.^20^, ^21^ Therefore, the first cases of Bt protein (Cry1F) resistance were detected after only a few years of commercialization.^19^, ^21^ Currently, Cry1F resistance is widespread in Brazil and Cry1F‐resistant larvae exhibit a high level of cross‐resistance to Cry1Ab, as well as maize hybrids expressing Cry1A.105/Cry2Ab, Cry1A.105/Cry2Ab2/Cry1F and Vip3Aa20/Cry1Ab.^22^, ^23^, ^24^

The fast evolution of resistance against Cry toxins has led to the need for additional insecticide applications during the cropping season.^25^ In southern Brazil, for example, up to four additional insecticide applications are required to control FAW.^25^ Therefore, it is essential to know the efficacy of chemical insecticides which could control Cry1F‐resistant individuals present in the refuge areas, using the best synergistic approach combining Bt technology and the rotation of effective insecticides. Hence, a better understanding of insecticide susceptibility of field populations as well as involved mechanisms of resistance is important for the implementation of sustainable control strategies.

The most common mechanisms involved in insecticide (and Bt toxin) resistance are target‐site mutations and enhanced detoxification. ^26^, ^27^, ^28^ Target‐site mutations in the voltage‐gated sodium channel (VGSC), acetylcholinesterase (AChE) and ryanodine receptor (RyR) have been reported in FAW populations from Brazil highly resistant to pyrethroids, organophosphates and diamide insecticides, respectively.^11^, ^29^ Moreover, target site resistance in the ATP‐binding cassette transporter subfamily C2 (ABCC2), conferred by a two amino acid deletion (glycine and tyrosine – GY‐deletion) was linked to high levels of Cry1F resistance in a FAW strain (Sf_Des) collected in Brazil.^30^

In order to better understand possible multi/cross‐resistance patterns, we have examined the efficacy of different insecticide modes of action, including Bt proteins towards the previously described Cry1F‐resistant (Sf_Des) and a Cry1F‐susceptible (Sf_Bra) strain. Furthermore, the toxicological profile of the two FAW strains (Sf_Des and Sf_Bra) was characterized at the molecular and biochemical level. Transcriptomic RNA‐Seq analysis and the activity of major detoxification enzymes involved in the detoxification pathways, such as P450 enzymes, carboxylesterases (CE), glutathione S‐ransferases (GST) and uridine diphosphate‐glucosyltransferases (UGTs), were investigated. Results obtained here at both phenotypic and genotypic levels provide a better understanding of the detoxification process of FAW towards synthetic insecticides and Bt insecticidal proteins, and provide practical support for managing Cry1F‐resistant individuals in a high‐dose/refuge system.

## 2 MATERIAL AND METHODS

### 2.1 FAW strains and rearing

Two *S. frugiperda* strains, Sf_Bra (susceptible to Cry1F, collected in the state of São Paulo, 2005) and Sf_Des (field‐resistant to Cry1F, collected in *São* Desidério – Bahia, 2016), described previously by Boaventura *et al*. (2020),^30^ were sampled in maize‐growing regions in Brazil. Larvae were fed on an artificial diet based on wheat germ and soybean powder without exposure to any Bt protein or synthetic insecticides. Adults were fed with 10% (v/v) malt solution every second day. The insects were reared under controlled conditions (25 ± 1 °C, 55 ± 5% relative humidity).

### 2.2 Chemicals and insecticidal proteins

All chemicals and solvents used in this study were of analytical grade unless otherwise stated. The representative active ingredient of nine different mode of action classes were of analytical grade and used according to information given in Table S1. Bradford reagent and Bovine Serum Albumin (BSA) were purchased from Bio‐Rad (Hercules, CA, USA). The chemicals 1‐chloro‐2,4‐dinitrobenzene (CDNB), l‐glutathione reduced from *Saccharomyces cerevisiae* (GSH), glutathione oxidized, 1‐naphthyl acetate (1‐NA), 1‐naphthyl butyrate (1‐NB), Fast blue RR salt, NADPH, ethylenediaminetetraacetic acid (EDTA), 1,4‐dithiothreitol (DTT), Triton X‐100, Tween‐80 and 7‐benzyloxy‐4‐(trifluoromethyl)‐coumarin (BFC) were purchased from Sigma Aldrich (Munich, Germany). The artificial substrates 7‐benzyloxymethoxy‐4‐trifluoromethyl coumarin (BOMFC) and 7‐benzyloxymethoxy resorufin (BOMR) were purchased from Vivid®, Thermo‐Fisher Scientific (Carlsbad, MA, USA). The cOmplete™ EDTA‐free proteinase inhibitor was purchased from Roche (Merck, Darmstadt, Germany).

The insecticidal toxins were produced by *B. thuringiensis* or *Escherichia coli* recombinant strains and kindly provided internally by Bayer (Chesterfield, MO, USA). Cry1Ab (91% purity) was sent as purified trypsin‐activated protein in 50 mm sodium bicarbonate (pH 10.25), Cry1Ac (28.2% purity) as lyophilized material, Vip3Aa (100% purity) in 25 mm Tris–HCl, 0.25 m sodium chloride (NaCl) and 2 mm DTT (pH 8.0) buffer.

### 2.3 Dose–response bioassays with chemical insecticides and Bt proteins

Representative active ingredients (15 different active substances) belonging to nine different modes of action were used at concentration ranges given in Table S1. The insecticides were dissolved in 10% (v/v) acetone and 0.1% (v/v) aqueous Triton X‐100 solution and the serial dilutions made in 0.1% (v/v) aqueous Triton X‐100. The insecticide concentrations used varied from 722 to 0.01 ng cm^−2^ (Table S1) and a solution of 0.1% (v/v) aqueous Triton X‐100 without active ingredient served as a negative control.

Artificial diet was placed in a 12‐well plate (Greiner Bio‐One, Austria) (2 mL diet/well) and an automated purpose‐built spraying device was used to apply (12 μL/well) the different doses of insecticides in at least five different concentrations (Table S1 for concentration range). The bioassays with synthetic insecticides were conducted with 3rd instar larvae of strains Sf_Bra and Sf_Des, by adding a single larva per well on diet treated with insecticide and sealed with perforated foil. The larvae were assessed for mortality (including larvae showing symptoms of poisoning) at three (3DAT) and seven days (7DAT) after treatment. The bioassays for the insecticidal proteins were performed with neonate larvae (<24 h old) according to Boaventura *et al*. (2020)^30^ and mortality was scored 5DAT. All Bt proteins were diluted in 50 mm sodium carbonate buffer (pH 10.4) and 0.1% (v/v) aqueous Triton X‐ 100 according to the concentrations described in Table S1.

The bioassay was replicated at least three times, each replicate consisting of 12 larvae per concentration tested. All larvae were kept at 25 ± 1 °C, 55 ± 5% relative humidity, and 16 h:8 h, light:dark photoperiod. Larvae were considered alive when they still reacted to outward stimuli and classified as affected when showing growth inhibition (1/3 of control) or strong poisoning effect, such as incomplete ecdysis for larvae exposed to triflumuron. Assays were considered valid when control mortality was ≤10%.

### 2.4 Preparation of enzymes and protein quantification

Pools of ten larvae (3rd instar) each of Sf_Bra and Sf_Des were homogenized on ice using a plastic mortar and 500 μL of different buffers according to the enzymatic assay to be conducted. In brief, for crude preparations of P450 enzymes, 0.1 m potassium phosphate buffer (pH 7.6) containing 1 mm EDTA, 1 mm DTT, 200 mm sucrose and cOmplete™ EDTA‐free Protease Inhibitor Cocktail tablet was used. For CE activity, tissue was homogenized in 0.1 m sodium phosphate buffer (pH 7.6) containing 0.1% (v/v) Triton X‐100. For GST activity 50 mm HEPES buffer (pH 6.8) containing 0.1% (v/v) Tween‐80 was used for MCB as substrate and 50 mm Tris–HCl buffer (pH 7.5) for CDNB.

The microsomal fraction for the P450 monooxygenase activity assay was obtained by centrifugation of homogenate for 5 min at 5000×*g* and 4 °C. The pellet was discarded, and the resulting supernatant was centrifuged at 4 °C for 15 min at 15 000×*g* followed by a last ultra‐centrifugation step at 100 000×*g* for 60 min at 4 °C. The microsomal pellet was resuspended in 300 μL 0.1 m potassium phosphate buffer (pH 7.6), 1 mm EDTA, 1 mm DTT, 5% (v/v) glycerol and served as enzyme source. For CE and GST activity, the homogenates were centrifuged at 10 000×*g* and 4 °C for 5 min and the supernatant collected. Protein concentration was determined using Bradford reagent and BSA as a reference.

### 2.5 Cytochrome P450 monooxygenases

Cytochrome P450 activity was measured according to Stumpf and Nauen (2001) with slight modifications. Coumarins (BOMFC and BFC) and resorufin (BOMR) were used as model substrates and determined fluorometrically in a black flat‐bottom 384‐well plate format (Greiner, Essen, Germany). Each reaction consisted of 25 μL enzyme source (25 μg protein) and 25 μL of the substrate solution (50 μm of the substrate and 1 mm NADPH in 0.1 M potassium phosphate buffer pH 7.6). Control reactions without NADPH and enzyme were included. The reactions with BOMFC and BFC were incubated for 1 h at 25 °C at 300 rpm in the dark. The self‐fluorescent NADPH was removed by adding 50 μL stop solution [50% (v/v) DMSO: TRIZMA‐base buffer (pH 10), 5 mm glutathione oxidized, 4 U mL^−1^ glutathione‐reductase] into each well. After another 30 min of incubation, fluorescence was determined in an endpoint assay at the appropriate excitation/emission wavelength settings according to manufacturer instructions. For the resorufin substrate BOMR, reactions were carried out as described above, without the addition of stop solution. The fluorescent product formation was measured using a kinetic assay for 1 h at 25 °C, with measurements taken every 5 min. All reactions were run in triplicate from four biological replicates per strain and measured using a spectrofluorometer Tecan Spark (Tecan Group Ltd., Switzerland).

### 2.6 Carboxylesterase activity

Enzyme substrate was prepared as described in Section 2.4 and CE activity was measured according to Grant *et al*.^31^ with minor modifications. The substrate stock solution contained 100 mm of 1‐NA or 1‐NB dissolved in acetone and 100 μL was added to 9 mL of a filtered solution of 1.5 mm Fast blue RR salt prepared in 0.2 m sodium phosphate buffer (pH 6.0). To determine esterase activity, 10 μL diluted enzyme source (5 μg protein) and 90 μL substrate solution containing 1‐NA or 1‐NB (final concentration 1 mm) was added to each well of a transparent flat bottom 384‐well microplate (Corning, USA). Reaction without enzyme source served as control and each reaction was run in triplicate. The esterase activity was monitored over 10 min at 25 °C with readings taken every 1.5 min using a Tecan Spark (Tecan Group Ltd., Switzerland) microplate reader at 450 nm for both substrates. The average activity was obtained from ten biological replicates per strain.

### 2.7 Glutathione S‐transferase activity

The GST activity was measured according to Nauen and Stumpf^32^ using CDNB and GSH as substrates and adapted for 384‐well microplates (Corning) with minor modifications. Reactions consisted of 25 μL enzyme solution (20 μg protein) and 25 μL substrate solution (0.05 m HEPES buffer pH 6.8 containing 0.1% (v/v) Tween‐80; CDNB and GSH at 0.4 mm and 4 mm final concentration, respectively). Reactions were run in triplicate for five biological replicates per strain. The change in absorbance was measured continuously for 5 min at 340 nm, and 25 °C in spectrofluorometer Tecan Spark (Tecan Group Ltd, Switzerland).

Assessment of GST activity using MCB as a substrate was performed in flat‐black 384‐well microplates (Greiner, Essen, Germany). The total reaction volume was 50 μL per well, consisting of 25 μL enzyme source (20 μg protein) and 25 μL substrate buffer containing MCB (final concentration 0.4 mm) and reduced glutathione (final concentration 2 mm). Measurements were taken every 2 min at kinetic modus for 20 min at 25 °C using a spectrofluorometer Tecan Spark (Tecan) at emission and excitation wavelengths of 465 nm and 410 nm, respectively. The nonenzymatic reaction of CDNB/MCB with GSH measured without enzyme served as control.

### 2.8 RNA extraction, RNA‐Seq and cDNA synthesis

Total RNA was extracted from third instar larvae (pools of ten larvae, in total five biological replicates per strain) of Sf_Bra and Sf_Des with TRIzol® reagent (Invitrogen, USA) and followed by RNA purification using RNeasy® Plus Universal Mini Kit (QIAGEN, Germany) according to manufacturer's instruction including a DNA digestion step with DNase I (QIAGEN). The RNA was quantified by spectrophotometry (NanoQuant Infinite 200; Tecan) and its integrity verified by an automated gel electrophoresis system, according to the CM‐RNA method (QIAxcel RNA QC Kit v2.0; QIAGEN). Around one μg total RNA was sent to GENEWIZ (Leipzig, Germany) and the RNA quality was checked with an Agilent 2100 BioAnalyzer. Further, an mRNA poly(A) enriched library was prepared and 150‐bp paired‐end reads were generated with NovaSeq Illumina sequencing platform (Illumina Inc., CA, USA).

For quantitative reverse transcription polymerase chain reaction (RT‐qPCR) validation of the expression profile of selected genes, one μg total RNA was used in 20‐μL reactions for cDNA synthesis using the iScript cDNA synthesis kit (Bio‐Rad, USA), following the manufacturer's instructions for RT‐qPCR analysis.

### 2.9 RNA‐Seq transcriptomic analysis and single nucleotide polymorphism (SNP) identification

Clean reads were obtained from GENEWIZ (Leipzig, Germany) and the transcriptome assembly was accomplished using trinity v2.8.5 and transdecoder v5.3.0.^33^, ^34^ Transcripts were translated using a transdecoder v2.0.1 pipeline.^34^ First, longest open reading frames (ORFs) with minimal length 30 amino acids were extracted using the TransDecoder.LongOrfs tool using a universal genetic code. Homology of ORFs to known proteins was determined by NCBI‐blastp v2.3.0+ search against the SWISSPROT database and PFAM domain prediction using hmmer v3.1b22–4. The most likely predicted ORFs were selected using TransDecoder.Predict and the longest ORF for each transcript was retained. Proteins containing interpro‐domain IPR002018 (Carboxylesterase, type B) or IPR001128 (Cytochrome P450) were classified as carboxylesterases or cytochrome P450s, respectively.

A multiple sequence alignment of 125 protein sequences identified as P450 was performed using MUSCLE v3.8.31 and fasttree v2.1.5 to create a maximum‐likelihood tree using geneious v10.2.6.

Functional annotation and gene ontology (GO) term assignment of translated longest ORFs of *de‐novo* assembled transcripts was performed using blast2go v1.3.3. ^35^ Therefore, domains were predicted using interproscan v5.17‐56.0 ^36^ and genes were searched against Uniprot KB using NCBI‐BlastP v2.2.27.^37^ GO term enrichment analysis was performed on differentially regulated genes using the bioconductor package goseq v1.28.0.^38^

Transcript quantification was determined by pseudoalignment with kallisto v0.45.0^39^ and summarized at the gene level using tximport v1.12.3.^40^ The bioconductor DEseq2 package v1.16.1 ^41^ in the R v3.4.1 environment was used to identify differentially expressed genes.

A *P*‐adjusted value (P*adjust*) ≤0.01 indicated statistical significance and Log_2_‐fold changes (log_2_FC) of ≥1 and <1 marked up‐ and downregulation, respectively.

Sequences of VGSC and AChE were obtained from separate trinity/transdecoder assemblies of the Sf_Bra and Sf_Des samples, respectively. VGSC and AChE sequences were identified by BLAST comparison *versus* the public *Spodoptera litura* sequences XP_022824852.1 and AQQ79919.1, respectively. Multiple protein alignment of VGSC/AChE from Sf_Bra, Sf_Des strains, *S. litura* and the partial sequence of *S. frugiperda* pyrethroid resistant strain (KC435026.1) and *S. frugiperda* organophosphate resistant strain (KC435023.1) were performed for target‐site identification. Sequences were compared for the presence of T929I, L932F and L1014F target‐site mutations in the VGSC, numbered according to *Musca domestica* sodium channel (GenBank X96668), and A201S, G227A and F290V in the AChE, numbered according to *Torpedo californica* (PDB ID: 1EA5).

### 2.10 Quantitative reverse transcription polymerase chain reaction (RT‐qPCR) for gene expression validation

Eleven *CYP* genes previously described by Giraudo *et al*.^42^ and Nascimento *et al*.^13^ to be involved in insecticide detoxification were investigated in Sf_Bra and Sf_Des strains by RT‐qPCR. The ribosomal genes *rsp3A*, *L17*, and *L10* were used as reference genes (primers and accession numbers of all genes are listed in Table S2). Reactions were performed using SsoAdvanced™ Universal SYBR® Green Supermix (Bio‐Rad, USA) according to the manufacturer's protocol. Briefly: reaction mixtures (10 μL) contained 2.5 μL cDNA (5 ng), 5 μL SsoAdvanced™ Universal SYBR® Green Supermix (Bio‐Rad), 400 nm of reverse/forward primers (Table S2), and nuclease‐free water. Reactions were run in triplicate using CFX384™ Real‐Time system (Bio‐Rad) and nontemplate mixtures as negative controls. The PCR conditions were: 3 min at 95 °C followed by 40 cycles of 95 °C for 15 s and 60 °C for 30 s. A final melting‐curve step was included post‐PCR (ramping from 65 °C to 95 °C by 0.5 °C every 5 s) to check for nonspecific amplification. Amplification efficiencies were determined by a five‐fold dilution series revealing for all primers an efficiency ≥93%.

### 2.11 Statistical analysis

Bioassay data considering dead and affected insects were fitted by a logistic regression model to calculate the EC_50_ values and 95% confidence intervals (prism v8, GraphPad Software Inc., CA, USA). Resistance ratios (RR) were estimated by dividing the EC_50_ value obtained for Sf_Des by the EC_50_ value of the susceptible strain (Sf_Bra).

The mean kinetic velocity was calculated as the increase of RFU/OD min^–1^ in the linear phase of the enzymatic reaction. Average enzyme activity obtained from Sf_Bra and Sf_Des were statistically compared by an unpaired Student's *t*‐test for each enzyme, substrate and larval development stage separately using prism v8.

The expression values obtained by RT‐qPCR were normalized to the reference genes and Sf_Des expressions were compared to Sf_Bra and analysed for statistical differences at *P* < 0.05 by Student's *t*‐test, with qbase ^+^ v3.2software (Biogazelle, Belgium).

## 3 RESULTS

### 3.1 Bioassays

The efficacy against FAW larvae of 12 different synthetic insecticides was tested and evaluated at 3DAT and 7DAT. As not much difference was observed between the two assessments, the full set of log‐dose mortality data for 7DAT is provided in Table 1 and for 3DAT in Table S3. The bioassay results indicate that the Cry1F‐resistant strain Sf_Des also developed significant resistance against deltamethrin (RR_50_ = 14‐fold) and chlorpyrifos (RR_50_ = 8‐fold).

**Table 1:** Log‐dose mortality data obtained for 12 different insecticides against 3rd instar larvae of *Spodoptera frugiperda* strains Sf_Des and Sf_Bra in diet spray bioassays. The assessment for affected larvae was made seven days after treatment

Compound	Strain	*n*	EC_50_ (ng ai cm^−2^)	95% CI^†^	Slope (±SE)	RR^‡^
Deltamethrin	Sf_Bra	324	0.20	0.18–0.22	2.47 (0.64)	
	Sf_Des	324	2.86	1.81–4.51	1.76 (0.44)	14.23
Chlorpyrifos	Sf_Bra	288	11.67	7.47–18.22	5.07 (1.48)	
	Sf_Des	288	92.58	64.85–132.20	2.40 (0.71)	7.93
Triflumuron	Sf_Bra	252	2.20	1.57–3.07	3.46 (0.82)	
	Sf_Des	336	8.08	2.51–25.97	0.72 (0.20)	3.68
Thiodicarb	Sf_Bra	540	43.39	37.99–49.56	6.94 (1.01)	
	Sf_Des	288	105.20	76.04–145.5	1.72 (0.49)	2.42
Spinosad	Sf_Bra	396	5.13	4.08–6.45	1.60 (0.30)	
	Sf_Des	396	8.42	7.02–10.10	4.06 (0.92)	1.64
Emamectin Benzoate	Sf_Bra	401	0.03	0.02–0.03	2.89 (0.42)	
	Sf_Des	401	0.04	0.03–0.04	1.64 (0.27)	1.18
Abamectin	Sf_Bra	288	91.62	63.96–131.30	3.06 (1.02)	
	Sf_Des	252	104.50	76.95–141.80	1.80 (0.38)	1.14
Tetraniliprole	Sf_Bra	252	1.46	1.03–2.06	2.98 (1.90)	
	Sf_Des	252	1.65	1.23–2.20	2.57 (0.84)	1.13
Chlorfenapyr	Sf_Bra	401	23.97	14.20–40.45	1.62 (0.71)	
	Sf_Des	401	25.30	12.99–49.28	1.39 (0.58)	1.06
Chlorantraniliprole	Sf_Bra	252	0.37	0.18–0.78	1.22 (0.45)	
	Sf_Des	252	0.39	0.33–0.46	3.28 (0.46)	1.03
Flubendiamide	Sf_Bra	401	4.44	3.78–5.23	6.55 (1.66)	
	Sf_Des	401	4.17	3.71–4.67	4.04 (0.60)	0.94
Indoxacarb	Sf_Bra	252	4.08	3.77–4.42	4.60 (0.49)	
	Sf_Des	252	3.74	3.16–4.43	3.62 (0.86)	0.92

^†^ 95% confidence interval.

^‡^ Resistance ratio (EC_50_ of Sf_Des strain divided by EC_50_ of Sf_Bra).

Almost no variation in susceptibility (RR 1‐3) was seen for the individual diamide insecticides, indoxacarb, spinosad, thiodicarb, triflumuron and chlorfenapyr. However, low but significant differences in susceptibility (nonoverlapping CI 95%) were observed for thiodicarb and spinosad at 7DAT (Table 1).

Neonate larvae of strains Sf_Bra and Sf_Des also were subjected to Bt toxicity assays towards Cry1Ac, Cry1Ab and Vip3Aa. The results indicate that strain Sf_Des – known to be resistant to Cry1F^30^ – shows high cross‐resistance levels against Cry1Ac (>100‐fold) and Cry1Ab (>400‐fold), but not Vip3Aa when compared to the susceptible reference strain Sf_Bra (Table 2).

**Table 2:** Log‐dose mortality data obtained for insecticidal proteins from *Bacillus thuringiensis* against neonate (<24 h) larvae of *Spodoptera frugiperda* strains Sf_Des and Sf_Bra in diet overlay assays. The assessment for affected larvae was made five days after exposure

Bt protein	Strain	*n*	EC_50_ (μg ai cm^−2^)	95% CI^†^	Slope (±SE)	RR^‡^
Cry1F^§^	Sf_Bra	190	0.098	0.0811–0.1188	1.51 (0.17)	>490
	Sf_Des	190	>48.70	ND	ND	
Vip3Aa	Sf_Bra	288	0.005	0.0047–0.0056	2.23 (0.29)	1
	Sf_Des	288	0.005	0.0041–0.0051	2.01 (0.24)	
Cry1Ab	Sf_Bra	288	0.080	0.0273–0.1370	1.14 (0.37)	439
	Sf_Des	180	34.95	29.629–43.419	1.42 (0.20)	
Cry1Ac	Sf_Bra	288	0.274	0.2005–0.3664	2.34 (0.82)	111
	Sf_Des	180	30.50	18.507–66.071	0.49 (0.07)	

ND, not determined.

^†^ 95% confidence interval.

^‡^ Resistance ratio (EC_50_ of Sf_Des strain divided by EC_50_ of Sf_Bra).

^§^ Data obtained from Boaventura *et al*. 2020.

### 3.2 Activity of detoxification enzymes (P450, CE and GST)

Cytochrome P450 activity was determined in a fluorometric assay with BOMFC, BFC and BOMR as model substrates (Table 3). The highest activity was obtained with BOMR in strain Sf_Des (112.72 ± 15.58 RFU min^−1^ mg protein^−1^), which was significantly higher than in Sf_Bra (5.85 ± 4.67 RFU min^−1^ mg protein^−1^), representing a 19‐fold difference. The coumarin‐based substrate BFC also revealed significant differences in activity, albeit at a much lower level (1.6‐fold), although no difference was observed with BOMFC (Table 3).

**Table 3:** Comparison of enzyme activity obtained from mass homogenates (3rd instar) of *Spodoptera frugiperda* strains Sf_Bra and Sf_Des for the main detoxification enzymes, cytochrome P450‐dependent monooxygenase (P450), carboxylesterase (CE) and glutathione S‐transferase (GST) using different model substrates. Activities were statistically analyzed by Student's *t*‐test comparing Sf_Des and Sf_Bra mean values

Enzyme	*n*	Substrate	Strain	Enzyme activity mg^−1^ (±SE)^†^	Ratio^‡^
P450	4	BOMR	Sf_Bra	5.85 (4.67)	19.3
			Sf_Des	112.72 (15.58)*	
		BOMFC	Sf_Bra	30.00 (6.86)	1.5
			Sf_Des	46.05 (9.73)	
		BFC	Sf_Bra	112.11 (13.19)	1.6
			Sf_Des	179.00 (18.90)*	
CE	10	1‐NA	Sf_Bra	337.98 (34.95)	1.0
			Sf_Des	346.53 (85.45)	
		1‐NB	Sf_Bra	216.72 (36.62)	1.0
			Sf_Des	223.54 (46.56)	
GST	5	CDNB	Sf_Bra	6.20 (0.50)	0.9
			Sf_Des	5.80 (0.76)	
		MCB	Sf_Bra	1310.89 (124.86)	0.8
			Sf_Des	1157.70 (294.53)	

* Indicates significant differences (*P* < 0.05, unpaired Student's *t*‐test).

^†^ Enzyme activity is shown as OD min^−1^ mg^−1^ or RFU min^−1^ mg^−1^. Means in the column followed by * are significantly different (α = 0.05, unpaired Student's *t*‐test).

^‡^ Mean activity obtained for Sf_Des divided by the mean activity of Sf_Bra.

No significant difference in CE activity was observed between Sf_Bra and Sf_Des using 1‐NA and 1‐NB (*P* > 0.05, two‐tailed unpaired Student's *t*‐test) as artificial substrates (Table 3), with slightly higher CE activity obtained when using 1‐NA as substrate.

The average GST activity between strain Sf_Bra and strain Sf_Des did not differ significantly for both substrates, CDNB and MCB, tested (Table 3).

In conclusion, the enzyme activity measurements performed with different substrates suggest a significantly increased activity of P450 enzymes of resistant strain Sf_Des, whereas no significant differences were observed for CE and GST activities.

### 3.3 Transcriptomics and target‐site mutations

A total of 209.969 trinity transcripts and 118.013 total trinity ‘genes’ were obtained from the cDNA libraries (Table S4). The average contig and median contig length were 993 and 424, respectively (Table S4). Transcript quantification was determined by pseudoalignment (Table S5), merged on gene level and filtered for genes with cumulative abundance of more than ten across all samples. The transcriptome was deposited in the NCBI Sequence Read Archive database under BioProject PRJNA641764.

**Figure 1:**
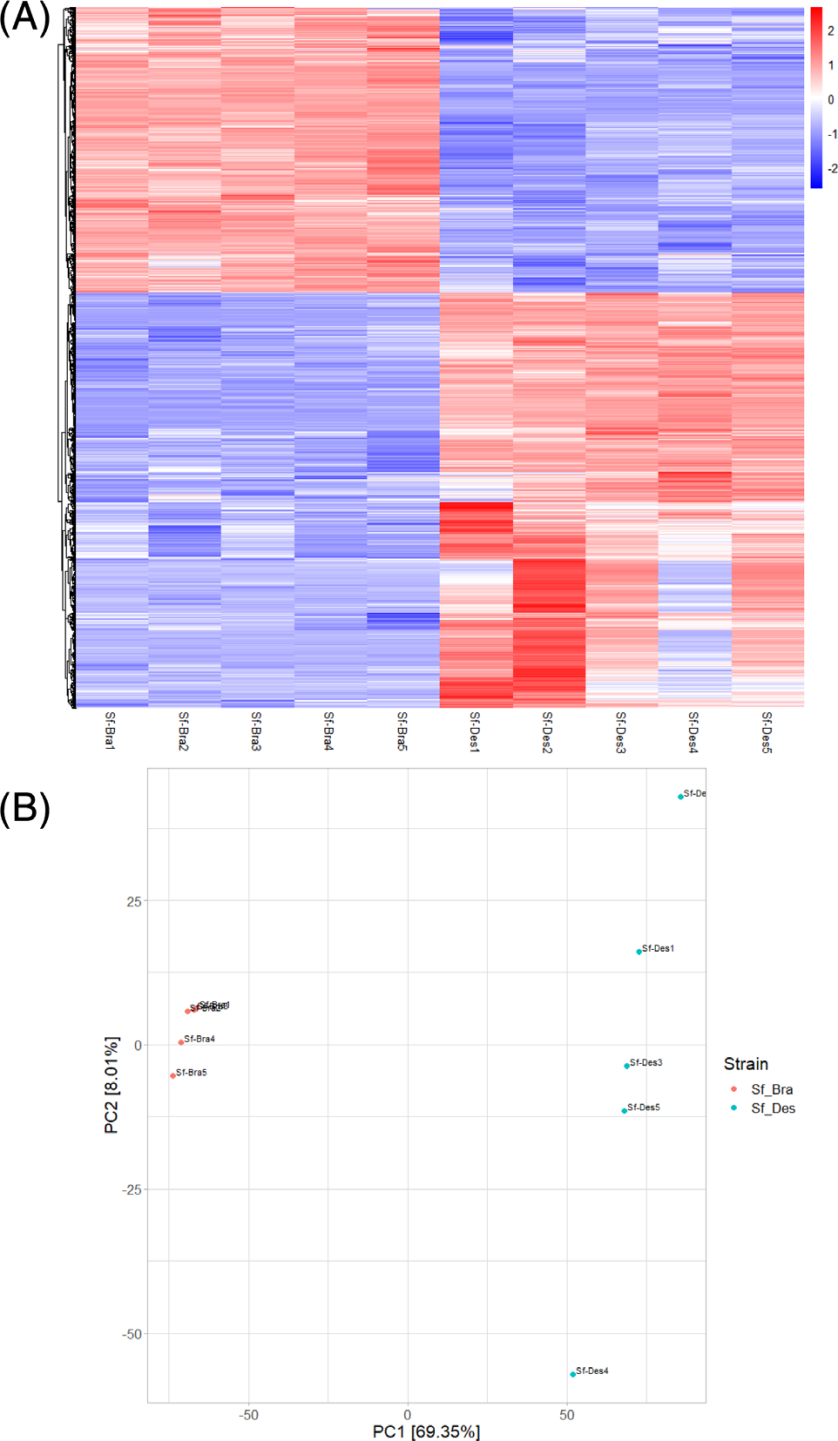
(A) Heatmap showing normalized differential expression level for the top 1460 genes (P*adjust* ≤ 0.01) between *Spodoptera frugiperda* strains Sf_Bra and Sf_Des based on minimal average expression across samples of 100 based on variance stabilizing transformation of DESEq2 package. (B) PCA of RNA‐Seq data obtained for strains Sf_Bra and Sf_Des.

**Figure 2:**
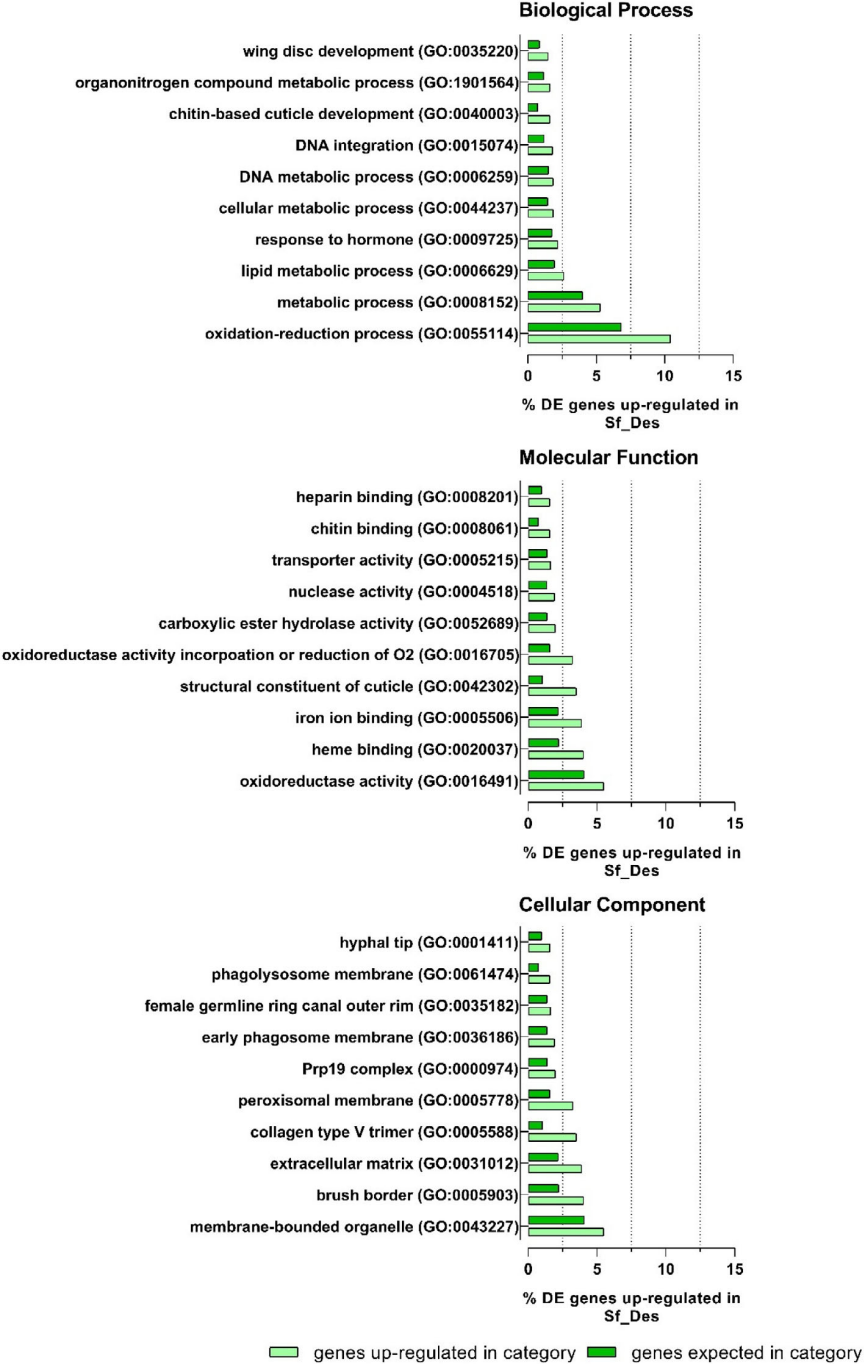
Overview of GO top ten categories and the respective percentage of differentially expressed transcripts (induced) of *Spodoptera frugiperda* Sf_Des strain in comparison to the susceptible reference strain Sf_Bra (P*adjust* < 0.01; DE in category ≥5) assigned to biological process, cellular component and molecular function.

A comparative gene expression analysis demonstrated that Sf_Des and Sf_Bra 3rd instar larvae have distinct gene expression profiles [Fig. 1(A)] and are well‐separated by principal component analysis [Fig. 1(B)]. Among the 57 534 genes evaluated, 12 339 were differentially expressed (P*adjust* ≤ 0.01, log_2_FC ≥ 1 and log_2_FC < 1) (Fig. S1). Functional annotation was performed with blast2go and GO terms could be assigned to 15 443 of 57 534 expressed genes. GO term enrichment of genes expressed at higher levels in Sf_Des revealed significant enrichment of 137 GO terms (P*adjust* ≤ 0.01, ≥5 regulated genes) distributed across all three GO domains (Biological Process: 74; Cellular Component: 12; Molecular Function: 51) (Fig. 2 and Table S6). The genes which showed the highest levels of overexpression related to detoxification processes (oxidation–reduction process, GO:0055114; metabolic process GO:008152) and cuticle development (chitin‐based cuticle development, GO:0040003) in Sf_Des (Fig. 2). This also is reflected by enrichment of GO terms in cellular localization (membrane‐bound organelle, GO:0043227; brush border, GO:0005903) and molecular functions (structural constituent of cuticle, GO:0042302, oxidoreductase activity, acting on paired donors, with incorporation or reduction of molecular oxygen, GO:0016705). In contrast, genes expressed more highly in Sf_Bra are enriched in 111 GO terms involved in DNA integration (GO:0015074) and transposition (GO:0032196) (Table S7).

The 43 transcripts with log_2_FC > 10 include genes involved in cuticle proteins, P450 enzymes (CYP9A‐like) and one aminopeptidase N‐like (Fig. S2). Conversely, five transcripts were highly downregulated in the Sf_Des strain log_2_FC < −10, described as zinc finger proteins and myrosinase.

The total number of transcripts assigned as P450, CE, GST, and UGT which were up‐ and downregulated in Sf_Des is shown in Table 4. Among the differentially expressed genes, the top ten candidate genes involved with detoxification pathways such as P450, CE, GST and UGT were selected and are displayed in Table 5.

**Table 4:** Differentially expressed genes (DEGs) potentially involved in *Spodoptera frugiperda* detoxification of synthetic insecticides. The DEGs listed have P*adjust* values of <0.01 and an absolute value of log_2_ FC ≥ 1 for upregulated genes and log_2_ FC < 1 for downregulated genes

Transcripts	Total	log_2_ FC ratio ≥ 1	log_2_ FC ratio < 1
Cytochrome P450 monooxygenase	125	85	40
Carboxylesterase	67	36	31
Glutathione S‐transferase	22	12	10
UDP‐glucosyltransferases	27	16	11

**Table 5:** List of top ten transcripts highly expressed in *Spodoptera frugiperda* Sf_Des strain for the main detoxification enzyme families

Transcript	Log_2_FC	Description
**P450**		
TRINITY_DN1680_c0_g2	12.62606	*Spodoptera litura* CYP9A39‐like
TRINITY_DN7188_c0_g1	10.84847	*Spodoptera litura* CYP9A39‐like
TRINITY_DN2295_c0_g1	10.56542	*Spodoptera frugiperda* CYP9A59
TRINITY_DN27386_c0_g1	9.147017	*Spodoptera litura* cytochrome P450 9e2‐like
TRINITY_DN36_c0_g1	9.144311	*Spodoptera litura* CYP9A39‐like
TRINITY_DN2295_c0_g3	8.144188	*Spodoptera frugiperda* CYP9A58
TRINITY_DN13552_c0_g1	8.058662	*Spodoptera litura* CYP9A40‐like
TRINITY_DN316_c2_g1	7.988559	*Spodoptera exigua* CYP6B31‐like
TRINITY_DN9618_c0_g1	7.874711	*Spodoptera litura* cytochrome P450 4d2‐like
TRINITY_DN18867_c0_g2	7.70106	*Spodoptera litura* CYP9A39‐like
**CE**		
TRINITY_DN14758_c0_g2	8.881116	*Spodoptera littoralis* antennal esterase CXE4‐like
TRINITY_DN32838_c0_g1	8.237679	*Papilio xuthus* epidermal growth factor receptor substrate 15‐like
TRINITY_DN13867_c0_g1	7.814419	*Spodoptera litura* esterase FE4‐like
TRINITY_DN15075_c0_g1	7.34096	*Spodoptera litura* juvenile hormone esterase‐like
TRINITY_DN12727_c0_g1	7.151613	*Spodoptera litura* esterase FE4‐like
TRINITY_DN11913_c0_g1	7.046612	*Spodoptera litura* acetylcholinesterase‐like
TRINITY_DN13577_c0_g2	5.215824	*Spodoptera litura* acetylcholinesterase‐like
TRINITY_DN40027_c1_g1	5.146553	*Spodoptera litura* juvenile hormone esterase‐like
TRINITY_DN28170_c0_g1	5.011122	*Spodoptera litura* juvenile hormone esterase‐like
TRINITY_DN1407_c0_g2	4.906461	*Spodoptera litura* esterase FE4‐like
**GST**		
TRINITY_DN12777_c0_g1	7.746027	*Spodoptera frugiperda* glutathione S‐transferase epsilon 9
TRINITY_DN16337_c0_g1	6.671198	*Spodoptera frugiperda* glutathione S‐transferase epsilon 9
TRINITY_DN28193_c0_g1	5.735641	*Spodoptera frugiperda* glutathione S‐transferase epsilon 14
TRINITY_DN22130_c0_g1	5.276331	*Spodoptera frugiperda* glutathione S‐transferase epsilon 14
TRINITY_DN6508_c0_g1	4.657795	*Spodoptera frugiperda* glutathione S‐transferase theta 1
TRINITY_DN32476_c0_g2	4.627718	*Spodoptera frugiperda* glutathione S‐transferase delta 1
TRINITY_DN28359_c0_g1	3.949156	*Spodoptera litura* glutathione S‐transferase‐like
TRINITY_DN977_c0_g2	3.398927	*Drosophila melanogaster* glutathione S‐transferase S1
TRINITY_DN30552_c0_g2	2.920379	*Manduca sexta* glutathione S‐transferase 1
TRINITY_DN2322_c0_g1	2.086194	*Spodoptera frugiperda* glutathione S‐transferase epsilon 12
**UGT**		
TRINITY_DN9608_c0_g2	10.6157	*Spodoptera litura* UGT 2B10‐like
TRINITY_DN10206_c0_g1	8.38444	*Spodoptera exigua* UGT 33F6 mRNA
TRINITY_DN10071_c0_g1	7.69253	*Spodoptera litura* UGT 2B31‐like
TRINITY_DN23825_c0_g1	7.10749	*Spodoptera litura* UGT 2B10‐like
TRINITY_DN31624_c0_g1	7.05726	*Spodoptera littoralis* UGT 40 L2‐like
TRINITY_DN22190_c0_g1	6.61785	*Spodoptera frugiperda* UGT 40D5
TRINITY_DN8387_c0_g1	6.20902	*Spodoptera litura* UGT 1‐7C‐like
TRINITY_DN1763_c2_g1	4.22764	*Spodoptera exigua* UGT 40F5‐like
TRINITY_DN29556_c0_g1	3.66796	*Spodoptera littoralis* UGT 40R3‐like
TRINITY_DN2969_c0_g1	3.15416	*Spodoptera exigua* UGT 33 V1‐like

**Figure 3:**
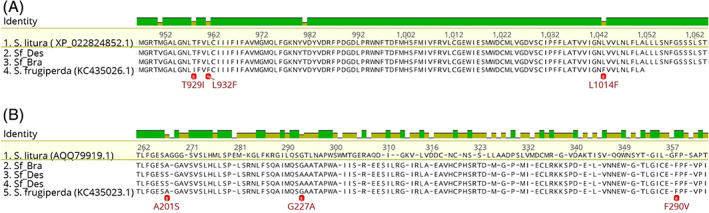
Sequences of VGSC and AChE were obtained from separate assemblies of the *Spodoptera frugiperda* Sf_Bra and Sf_Des strains and compared to *S. litura* sequences for VGSC (XP_022824852.1) and AChE (AQQ79919.1), and partial sequences of VGSC (KC435026.1) and AChE (KC435023.1) obtained from *S. frugiperda* strains resistant to pyrethroid and organophosphate, respectively. Sequences were compared for the presence of T929I, L932F and L1014F target‐site mutations in the VGSC, numbered according to *Musca domestica* sodium channel (GenBank X96668) and A201S, G227A and F290V in the AChE, numbered according to *Torpedo californica* AChE (PDB ID: 1EA5).

As our results indicated a high level of differential expression of *CYP* genes, an aligned and tree based on amino acid identity of P450 assigned transcripts was performed (Fig. S2). Highlighting the transcripts with log_2_FC > 5 revealed that most of them are grouped in close‐related branch in the cladogram and were annotated as *CYP9A*‐like genes (Fig. S3). On the one hand, alignment of the VGSC [Fig. 3(A)] and AChE [Fig. 3(B)] from consensus amino acid sequences obtained from five biological replicates of the Sf_Bra and Sf_Des strains, reference *S. litura* and *S. frugiperda* resistant strains revealed no target‐site mutation linked to pyrethroid resistance in the VGSC of Sf_Des and Sf_Bra. On the other, A201S and G227A mutations in the AChE (numbering according to *Torpedo californica*: PDB ID: 1EA5) were observed in Sf_Des.

### 3.4 RT‐qPCR

The RT‐qPCR analysis validated the RNA‐Seq data for 11 selected *CYP* genes. Among the genes tested, three were significantly upregulated in Sf_Des, *CYP9A*‐like and *CYP6B39* expression were up to 260‐fold higher, and *CYP9A59* was three‐fold‐overexpressed. however, *CYP321B1*, *CYP332A1*, *CYP321A2*, and *CYP9A28* were significantly downregulated in Sf_Des (Table 6).

**Table 6:** Validation of differentially expressed genes by RT‐qPCR analysis. The expression level of 11 genes representing genes involved in the metabolism of insecticides was investigated in the Cry1F‐resistant *Spodoptera frugiperda* strain Sf_Des by normalization to the expression of *RPS3A*, *L10* and *L17* and compared to the expression of Sf_Bra. Expressions were statistically analyzed with qbase+ software (unpaired Student's *t*‐test, *P* < 0.05). The average relative expression and their respective 95% CI were obtained from five biological replicates run in triplicates

Gene	Strain	Average relative quantity	95% CI high	95% CI low	Comparison (Sf_Des/Sf_Bra)	Statistics
*CYP9A‐like*	Sf_Bra	1.00	23.47	0.04		
	Sf_Des	267.18	385.44	185.20	267.18	*
*CYP6B39*	Sf_Bra	1.00	28.61	0.03		
	Sf_Des	257.60	370.30	179.19	257.60	*
*CYP9A59*	Sf_Bra	1.00	1.42	0.70		
	Sf_Des	3.37	5.22	2.17	3.39	**
*CYP321A9*	Sf_Bra	1.00	1.34	0.75		
	Sf_Des	1.41	1.92	1.03	1.41	ns
*CYP333B4*	Sf_Bra	1.00	1.21	0.82		
	Sf_Des	1.02	1.31	0.80	1.01	ns
*CYP6B50*	Sf_Bra	1.00	1.27	0.79		
	Sf_Des	0.82	1.29	0.52	0.82	ns
*CYP321‐like*	Sf_Bra	1.00	0.63	1.58		
	Sf_Des	0.62	0.48	0.81	0.62	ns
*CYP321B1*	Sf_Bra	1.00	1.65	0.61		
	Sf_Des	0.48	0.61	0.39	0.48	*
*CYP9A28*	Sf_Bra	1.00	1.80	0.55		
	Sf_Des	0.24	0.48	0.12	0.24	**
*CYP332A1*	Sf_Bra	1.00	1.36	0.73		
	Sf_Des	0.12	0.14	0.10	0.12	***
*CYP321A7*	Sf_Bra	1.00	1.52	0.66		
	Sf_Des	0.07	0.12	0.04	0.07	***

ns, not significantly different.

^*^ *P* < 0.05.

^**^ *P* < 0.01.

^***^ *P* < 0.001 (unpaired Student's *t*‐test).

## 4 DISCUSSION

The fast evolution of resistance to many synthetic insecticides ^10^, ^11^, ^12^, ^13^, ^14^ and insecticidal proteins ^21^, ^22^, ^24^, ^43^ challenges the control of FAW in Brazil. The increase in resistance is a result of strong selective pressure by frequent sprays with insecticides belonging to a few modes of action and high adoption of Bt crops with low compliance to refuge areas.^19^ Therefore, it is of utmost importance to understand the overall toxicological profile of representative strains of resistant insects and their cross‐resistance patterns.

The susceptibility of a Cry1F‐resistant (Sf_Des) and Cry1F‐susceptible strain (Sf_Bra) was assessed against several chemical insecticides and Bt proteins that are currently used and expressed in maize, cotton and soybean to control FAW in Brazil. Very recently we provided a detailed analysis of the mechanistic and genetic basis of Cry1F resistance in strain Sf_Des.^30^ We described a GY‐deletion in ABCC2 which confers high levels of Cry1F resistance and this mutant was shown to be widespread in Brazil – thus qualifying strain Sf_Des as a surrogate to investigate patterns of susceptibility and potential cross‐resistance issues associated with the presence of the observed mutation. We compared the larval transcriptome of strains Sf_Bra and Sf_Des as a step towards understanding the molecular mechanisms possibly influencing the toxicological profile obtained by insecticide bioassays and the detoxification activity obtained in the biochemical assays to support the development of efficient insecticide resistance management strategies.

Although target‐site mutations can have a direct effect on the susceptibility to compounds targeting the same receptor,^11^, ^44^ metabolic resistance can affect a much broader range of compounds.^45^, ^46^, ^47^ FAW adaptation to cope with many different plant allelochemicals, is driven by detoxification systems including cytochrome P450s, CE, GST, UGTs and oxidative stress genes which were shown to support its ability to detoxify a broad range of insecticides.^42^, ^48^, ^49^, ^50^, ^51^

Moderate resistance ratios in strain Sf_Des were found for the pyrethroid deltamethrin (RR_50_ = 14‐fold). In Brazil, pyrethroid resistance has been reported towards *lambda*‐cyhalothrin (18‐fold)^11^ and shown to be conferred by target‐site mutations in the VGSC (T929I, L932F and L1014F), as well as significantly upregulated GST gene expression.^11^

Our transcriptome analysis revealed that 85, 36, 12 and 36 transcripts belonging to P450s, CE, GST and UGTs, respectively, were upregulated in Sf_Des. The enzymatic assay conducted with BOMR in this study showed that P450 activity is significantly higher in Sf_Des and supported by elevated expression levels of *CYP9A*‐like, *CYP6B39* (>200‐fold), and *CYP9A59* (three‐fold) as shown by RT‐qPCR analysis. The use of fluorescent model substrates is a common methodology to quantify the activity of P450 enzymes.^52^, ^53^ However, differences in substrate specificity need to be considered and as shown here, BOMR was the substrate showing the highest activity in Sf_Des when compared to the reference strain Sf_Bra. Moreover, the comparison of the VGSC sequence obtained for Sf_Bra and Sf_Des revealed the absence of commonly known target‐site mutations and suggests a metabolic mechanism conferring pyrethroid resistance in strain Sf_Des. This is supported by the detected overexpression of some of the P450s mentioned above. High expression levels exceeding several 100‐fold of individual P450s such as CYP6BQ23 were recently shown to confer pyrethroid resistance in pollen beetle (*Meligethes aeneus*).^53^

However, further bioassays with P450 inhibiting synergists and functional validation of the highly expressed candidate P450 genes in follow‐up studies is essential to investigate the oxidative detoxification potential towards pyrethroids.

Our bioassays revealed a decrease in susceptibility towards chlorpyrifos (eight‐fold) by comparing EC_50_ values obtained at 7DAT. Resistance towards chlorpyrifos has been described for FAW collected in Brazil,^11^ and associated with an increase of CE and/or GST activities and target‐site mutations in the AChE (A201S, G227A and F290V).^8^, ^11^, ^54^, ^55^ Although the transcriptome analysis did show that few GSTs and CE were upregulated in Sf_Des, no significant differences in activity were detected with the substrates tested. The AChE sequence comparison revealed the presence of the mutations A201S and G227A in Sf_Des which could explain the resistance level observed. Heterologous expression of AChE wild‐type from the silkworm (*Bombyx mori*) and AChE harboring the mutations A303S, G329A and L554S suggest reduction in AChE sensitivity to carbamate and organophosphate insecticides.^56^

For all other compounds tested throughout this study EC_50_ values obtained for Sf_Des did not differ significantly from Sf_Bra, except for a few cases with negligible levels of resistance, such as thiodicarb (2.4‐fold) and spinosad (1.6‐fold). Nevertheless, resistance to diamide insecticides has been observed in a laboratory‐selected strain carrying the I4734M mutation in the RyR and underpins the potential of FAW to develop diamide resistance under field conditions.^29^ Resistance to spinosyns was described in FAW in Brazil,^13^, ^14^ but our study revealed a lack of resistance in Sf_Des. Also, a low level of resistance was recorded for the carbamate thiodicarb for Sf_Des supporting the recommendation of this compound for soybean seed treatment to control early damage by FAW.^57^ Chlorfenapyr also has shown a lack of resistance towards the Sf_Des strain, confirming the results recently published for FAW from Brazil.^58^ Chlorfenapyr is a pro‐insecticide, which has to be activated by P450 enzymes.^59^ Therefore, the hypothesis that the overall high activity of P450 enzymes might contribute to chlorfenapyr toxicity even in resistant insects, has been considered previously.^58^, ^60^ Indoxacarb also is a pro‐insecticide, yet it is activated by esterases through cleavage of the *N*‐carbomethoxy group, resulting in an active metabolite that potently blocks the VGSC.^61^

Our bioassay results with different Bt proteins showed excellent control of the Cry1F‐resistant strain Sf_Des with Vip3Aa. On the one hand, the Vip3Aa protein does not share binding sites with the Cry1 proteins and therefore crops expressing Vip3A alone or combined with other Cry proteins were shown to effectively control Cry1F‐resistant *S. frugiperda*.^62^, ^63^ On the other, there is a high level of cross‐resistance among Cry1 proteins in *S. frugiperda*,^24^, ^64^ as confirmed by our results for Cry1Ab (RR >400‐fold) and Cry1Ac (RR >100‐fold). Therefore, gene‐pyramiding of two or more dissimilar Bt proteins is preferred to delay insect resistance.^65^

Recently, a midgut transcriptome analysis was performed with *S. exigua* exposed to sublethal doses of Cry1Ca and among the DEG, some differences in expression of P450, CE and GST genes were observed besides Bt‐related genes, such as ABC transporters.^66^ Moreover, cross‐resistance between pyrethroids and Cry1Ac has been reported in the diamondback moth, *Plutella xylostella*. Genetic studies suggest that possible interactions between esterases and Bt protein and/or indirect triggering of a defense metabolic pathway are involved and genetically linked at a common locus.^67^ Likewise, Gunning *et al*.^68^ have shown by *in vivo* assays that esterases from *Helicoverpa armigera* can bind to Cry1Ac, indicating that esterases may play a versatile role in resistance development to both Bt and conventional insecticides.^48^, ^69^ Moreover, Zhu *et al*.^70^ detected a co‐development of multiple or cross‐resistance to both organophosphate insecticides and Cry1F toxin in FAW, supported by high CE and GST activities in the Cry1F‐resistant strain. However, in our studies no significant difference in CE or GST activities could be detected between Sf_Bra an Sf_Des.

The CYPs have been observed to respond to sublethal doses of Cry toxins in different insect species, such as *Choristoneura fumiferana*, *Manduca sexta*, *Ostrinia nubilalis* and also *S. exigua*.^66^, ^71^, ^72^ In *S. exigua*, *CYP4S9*, *CYP6AB31*, *CYP6AE47* and *CYP9A* were upregulated after exposure to Cy1Ca and a similar response was observed in insects exposed to insecticides (*lambda*‐cyhalothrin, chlorantraniliprole, metaflumizone and indoxacarb).^66^, ^73^ A few studies have shown that CE is related to Cry resistance in *P. xylostella*, *O. furnacalis* and *C. medinalis* ^74^, ^75^, ^76^ and GST were downregulated in *O. furnacalis* to Cry1Ab and *C. medinalis* to Cry1Ac, Cry1Ab and Cry1C. ^76^, ^77^ In our studies, we have not checked the expression pattern after the exposure to Bt proteins. However, very high constitutive expression of *CYP9A*‐like and *CYP6B39* (> 200‐fold) were observed in Sf_Des, suggesting a role of P450 in general detoxification.

Giraudo *et al*.^42^ have shown that all members of the CYP9A subfamily are detected in the midgut, fat body, and Malpighian tubules and showed a response to sublethal doses of insecticides. A *CYP9A*‐like gene also was found to be upregulated in FAW resistant to lufenuron,^78^ supporting the association between CYP9A and insecticide resistance. Likewise, *CYP6B39* was the gene upregulated by most compounds tested, including insecticides and plant allelochemicals.^42^ As samples here were taken from the whole body, we cannot disregard the fact that genes belonging to the same subfamily can have tissue‐specific expression, for instance, *CYP6AE44* was not detected in the midgut and Malpighian tubules but was present in the fat body in FAW.^42^

Recently, the variation of gene copy number in a locus which includes a cluster of P450 genes has been described to play an important role in insecticide resistance ^79^ and host‐plant range in *S. frugiperda*.^80^ *CYP9A* genes were overexpressed upon the treatment of insecticides ^42^ and were found in two copies clustered together with alcohol dehydrogenase in resistant FAW populations from Puerto Rico.^79^

In our experiments, *CYP321A1* and *CYP321A7* were significantly downregulated in Sf_Des, whereas expression of *CYP321A9* did not differ from Sf_Bra. However, in *Helicoverpa zea* CYP321A1 has been shown to metabolize plant toxins such as xanthotoxin as well as insecticides including aldrin, cypermethrin and diazinon.^81^, ^82^ The next step would be to functionally validate the role of CYP9A‐like and CYP6B39 enzymes in FAW and the detoxification of insecticides by their recombinant expression.

The diversity of UGTs in lepidopterans also has indicated their contribution to the process of detoxification through glycosylation^83^, ^84^ and there are several indications in which an increase in the expression level of UGTs have been related to resistance to insecticides such as DDT.^85^, ^86^ In strain Sf_Des studied here, 16 UGT related genes were upregulated and are candidates for a more detailed investigation.

Results of this study, in conjunction with those reported elsewhere ^8^, ^87^, ^88^ demonstrate that FAW insecticide resistance is conferred by multiple biochemical and molecular mechanisms, although most of the chemical classes of insecticides, except two – pyrethroids and organophosphates – worked well against a Brazilian strain highly resistant to Cry1 toxins, suggesting that the GY‐deletion in ABCC2 conferring Cry1F resistance in Sf_Des does not result in significant resistant issues towards many chemical classes of insecticides. The high expression levels associated with many genes encoding detoxification enzymes, mainly P450s, even in the absence of insecticide pressure underpins the constitutive nature of the overexpression in strain Sf_Des compared to Sf_Bra, a strain maintained under laboratory conditions for 15 years. The metabolism of insecticides in insects certainly involves a series of complex metabolic processes and there are important gaps such as the multiple roles of detoxification enzymes related to the physiological and molecular mechanisms that control the processes of detoxification.^89^, ^90^

Our study provides a global transcriptomic profile with special emphasis on detoxification genes in a Bt‐resistant Brazilian FAW strain and identified candidate genes to explore further regarding their role in insecticide metabolism. Our results support the significant difference between Sf_Des and Sf_Bra in expression and activity of P450 genes possibly involved in xenobiotic (including insecticide) metabolism, which could support some of the phenotypical resistance observations in the bioassays (e.g. against pyrethroids in the absence of target‐site mutation). However, our study does not suggest cross‐resistance to many synthetic insecticides in strain Sf_Des shown to be highly resistant to Cry1 toxins conferred by a mutation in ABCC2.

**Figure 4:**
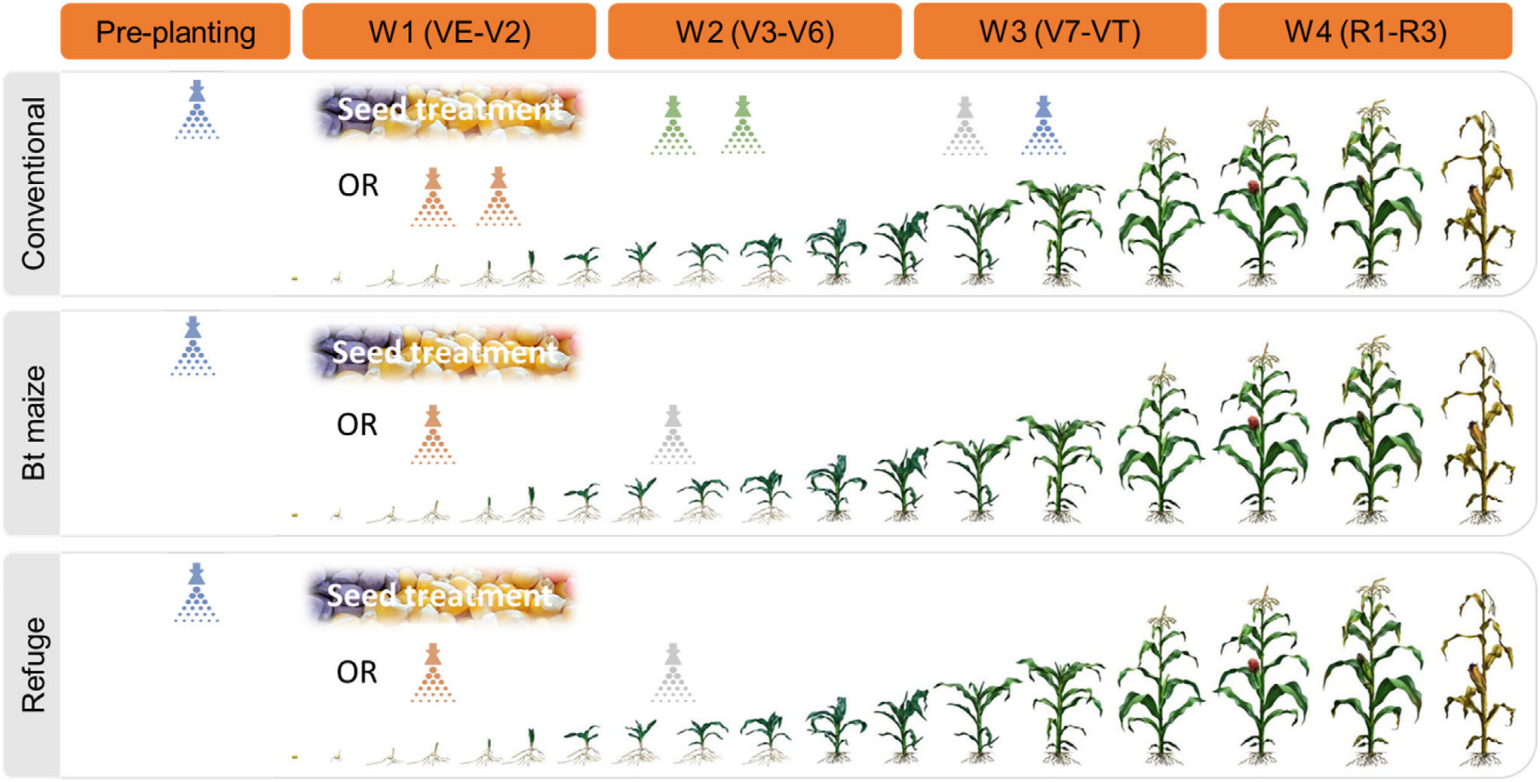
Proposed insecticide application scheme to control fall armyworm (FAW), *Spodoptera frugiperda*, through a growing season that avoids treating consecutive FAW generations with the same mode of action (MoA) in conventional maize, maize expressing *Bacillus thuringiensis* (Bt) insecticidal proteins and refuge areas. The shown schemes are based on the ‘MoA treatment windows’ approach recommended by IRAC and aim to manage FAW by different MoA in windows representing the mean duration of a single generation (30 days). Each ‘spray’ color represents a different MoA according to the IRAC MoA classification. Multiple applications of the same MoA are possible within a treatment window. When a treatment window is completed, a different MoA should be selected for use in the next 30 days, and if possible, a different MoA should even be applied in a third MoA treatment window. The example shown is based on a situation with four different MoA´s available and working equally good against FAW.

The use of chemical insecticides in refuge areas should be chosen and rotated based on insecticides with good efficacy against Bt‐resistant insects, such as those identified here (e.g. triflumuron, thiodicarb, chlorfenapyr, emamectin benzoate, indoxacarb and diamides). A typical resistance management scenario as recommended by IRAC is proposed in Fig. 4 in alignment with the so‐called ‘mode‐of‐action treatment windows approach’ to ensure that successive generations of the pest are not exposed to the same insecticide or insecticides showing cross‐resistance through a growing season. Moreover, pyramided maize expressing Bt toxins with low cross‐resistance to Cry1F might be preferred. Therefore, the results presented here for chemical and Bt‐based insecticides have important implications for resistance management in Bt crops and IPM programs.

## Supporting information

**Appendix S1.** Supporting information
