# Supplementary material for: Toxicological and molecular profiling of insecticide resistance in a Brazilian strain of fall armyworm resistant to Bt Cry1 proteins
Source: Pest Manag Sci. 2020 Sep 10;77(8):3713–26. doi: 10.1002/ps.6061 (PMC8359450; doi:10.1002/ps.6061)
Supplement: Supplementary file 1 — Appendix S1. Supporting information [file PS-77-3713-s001.docx]

**Toxicological and molecular profiling of insecticide resistance in a Brazilian strain of fall armyworm resistant to Bt Cry1 proteins**

Running title: Toxicological and molecular profiling of FAW resistance

Debora Boaventura,^a,b^ Benjamin Buer,^b^ Niklas Hamaekers,^b^ Frank Maiwald,^b^

and Ralf Nauen^b^*

^a^ Institute of Crop Science and Resource Conservation, University of Bonn, Bonn, Germany

^b^ Bayer AG, Crop Science Division, R&D Pest Control, Monheim, Germany

*Correspondence to:

Ralf Nauen, Bayer AG, R&D Pest Control, Building 6260, Alfred Nobel Str. 50, D-40789 Monheim, Germany. E-mail: [ralf.nauen@bayer.com](mailto:ralf.nauen@bayer.com)

**Supplementary data**

**Table S1** Overview on insecticides and insecticidal proteins used in the bioassays, including their mode of action according to the IRAC (Insecticide Resistance Action Committee) classification scheme and their respective dose range used in diet bioassays.

**Table S2** List of primers used for validation of differentially expressed genes by RT-qPCR analysis of Sf_Des and Sf_Bra *Spodoptera frugiperda* strains.

**Table S3** Log-dose mortality data obtained for 12 different insecticides against 3^rd^ instar larvae of Sf_Des and Sf_Bra in diet spray bioassays. The assessment for affected larvae was made three days after treatment.

**Table S4** Summary of RNA-Seq analysis obtained from 3^rd^ instar larvae of *Spodoptera frugiperda* strains Sf_Des and Sf_Bra (n=5 per strain).

**Table S5** Summary of transcript quantification obtained from 3^rd^ instar larvae of *Spodoptera frugiperda* strains Sf_Des and Sf_Bra by pseudoalignment with kallisto v0.45.0 [44] and summarized on gene level using tximport v1.12.3.

**Table S6** Gene Ontology (GO) term enrichment analyses of differentially expressed genes (DEG) that are up-regulated in Cry1F-resistant strain Sf_Des (P*adjust* < 0.01 and DEG ≥ 5 in each category).

**Table S7** Gene Ontology (GO) term enrichment analyses of differentially expressed genes (DEG) that are down-regulated in Cry1F-resistant strain Sf_Des (P*adjust* < 0.01 and DEG ≥ 5 in each category).

**Figure S1** Volcano plot of overall expression level of transcripts obtained for pooled 3^rd^ instar larvae of *Spodoptera frugiperda*.

**Figure S2** Differentially expressed genes (P*adjust* ≤ 0.01) obtained from *Spodoptera frugiperda* strains Sf_Des and Sf_Bra. Sf_Des is highly resistant to Cry1F insecticidal protein and Sf_Bra is the susceptible reference strain.

**Figure S3** Maximum-likelihood tree built by FastTree 2.1.5 (Geneious v.10.2.6) from multiple sequence alignment of 125 protein sequences identified as P450. Transcripts with log_2_FC >5 (n=37) are highlighted in blue as well as the monophyletic group encompassing CYP9-like transcripts.

**Table S1**

| **IRAC group** | **Mode of action** | **Chemical class** | **Active ingredient** | **Manufactory (Purity %)** | **Insecticide range (ng cm^-2^)** |
| --- | --- | --- | --- | --- | --- |
| IRAC 1A | AChE^2^ inhibitor | Carbamate | Thiodicarb | Sigma-Aldrich (99.2) | 0.05 - 722 |
| IRAC 1B |  | Organophosphate | Chlorpyrifos | Sigma-Aldrich (97.5) | 0.05 - 722 |
| IRAC 3A | VGSC^3^ modulator | Pyrethroids | Deltamethrin | Sigma-Aldrich (99.8) | 0.02 - 722 |
| IRAC 5 | nAChR^4^ allosteric modulators - site I | Spinosyns | Spinosad | Sigma-Aldrich (95.0) | 0.02 - 144 |
| IRAC 6 | GluCl^5^ allosteric modulator | Avermectins | Emamectin | Sigma-Aldrich (99.6) | 0.02 - 5.70 |
|  |  |  | Abamectin | Sigma-Aldrich (97.9) | 0.23 - 722 |
| IRAC 11 | Disruptors of insect midgut | *Bacillus thuringiensis* | Cry1Ab | Bayer AG (91.0) | 7.5 - 40,000 |
|  |  |  | Cry1Ac | Bayer AG (28.3) | 7.5 - 40,000 |
|  |  |  | Vip3Aa | Bayer AG (100) | 0.03 - 200 |
| IRAC 13 | Uncoupler of oxidative phosphorylation | Pyrroles | Chlorfenapyr | Dr. Ehrenstorfer (99.6) | 0.23 - 722 |
| IRAC 15 | Inhibitors of chitin biosynthesis affecting CHS1^6^ | Benzoylureas | Triflumuron | Bayer AG (99.6) | 0.05 - 722 |
| IRAC 22 | VGSC^3^ blockers | Oxadiazines | Indoxacarb | Sigma-Aldrich (99.5) | 0.23 - 722 |
| IRAC 28 | RyR^7^ modulators | Diamides | Tetraniliprole | Bayer AG (98.0) | 0.23 - 28 |
|  |  |  | Flubendiamide | Sigma-Aldrich (98.0) | 0.23 - 144 |
|  |  |  | Chlorantraniliprole | Sigma-Aldrich (98.0) | 0.01 - 28 |

**Table S2**

| **Primers** | **GenBank Accession number** | **Forward primer (5' to 3')** | **Reverse primer (5' to 3')** |
| --- | --- | --- | --- |
| *Sf_RPS3A*^1^ | AF429977 | CAACTCTGAACTTCGTGAG | CTACCACCCTCTCCATGAA |
| Sf_L10^2^ | MN044628 | GTCGTGCCAAGTTCAAGTTC | GTCCTCACGCAGCTTCTC |
| Sf_L17^2^ | MN044629 | GTGACGGAAGCTATCAAGAC | ACTTGTTGCCGAGGACAC |
| Sf_CYP321A1-like^4^ | PRJNA299878^5^ | CAAACCAGCCTGCACCTGTA | GGGCAACAGGACGTGTATAGG |
| Sf_CYP321B1^3^ | KC789754 | CGTACGATGCAGTCTTGGAA | CATTGCCTACAGGCAGAACA |
| Sf_CYP321A7^3^ | KC789750 | TCCAGACCCAGAAGTTTTCG | CGGCCTGGACTTGTAATTTG |
| Sf_CYP321A9^3^ | KC789752 | GCGTGGTGTAGCCTTCTACG | CGGGTCAATGACAAACAGTG |
| Sf_CYP9333B4^3^ | FP340412.1 | GAATTATGCCGGTGGTGTCT | TAGCGACATGTCTCGGTGAG |
| Sf_CYP332A1^3^ | FP340417.1 | GCATGCATGAAACGCTAAGA | CCACGTTCACGTAGACTGGA |
| Sf_CYP6B39^3^ | FP340416.1 | AAGTTCCAAGTGGAGCCATCGAGG | CCTCCTTTGGGCCCGACGAGAAG |
| Sf_CYP6B50^3^ | KC789749 | CAATCCAGCACGATGAGAAA | GTGCGAATTTTGACCAAGG |
| Sf_CYP9A9-like^4^ | PRJNA299878^6^ | CAATGCAATTCCTTGGACCAA | GCACACCATCGATCCAATGA |
| Sf_CYP9A28^3^ | FP340410.1 | TCAAGCACATCAAGCCAGTC | CCGTTGTGAGTCCATCACTGAC |
| Sf_CYP9A59^3^ | KJ671578 | GGATACCCACGTATGCCATC | TCCTAGGACCAGTGCCAAAT |
| Primer pairs described by^1^ Li et al. (2017); ^2^ Boaventura et al. (2020); ^3^ Giraudo et al. (2015);^4^Nascimento et al. (2015);^5^ Under the SRA accession number PRJNA299878 in Nascimento et al. (2015) named as transcript L_669_T_9/12; ^6^Under the SRA accession number PRJNA299878 in Nascimento et al. (2015) named as transcript L_464_T_3/3. | | | |

**Table S3**

| **Compound** | **Strain** | **n** | **EC_50_**  **(ng ai cm^-2^)** | **95 % CI^a^** | **Slope (±SE)** | **RR^b^** |
| --- | --- | --- | --- | --- | --- | --- |
| Deltamethrin | Sf_Bra | 324 | 0.20 | 0.18 - 0.22 | 2.47 (0.52) |  |
|  | Sf_Des | 324 | 3.49 | 2.37 - 5.14 | 2.74 (0.87) | 17.44 |
| Chlorpyrifos | Sf_Bra | 288 | 10.68 | 7.99 - 14.27 | 4.62 (1.00) |  |
|  | Sf_Des | 288 | 80.22 | 62.69 - 102.70 | 2.78 (0.48) | 7.51 |
| Triflumuron | Sf_Bra | 252 | 1.39 | 0.61 - 3.16 | 2.50 (0.63) |  |
|  | Sf_Des | 336 | 6.28 | 4.88 - 8.13 | 0.77 (0.06) | 4.53 |
| Emamectin Benzoate | Sf_Bra | 401 | 0.06 | 0.05 - 0.07 | 2.79 (1.42) |  |
|  | Sf_Des | 401 | 0.19 | 0.05 - 0.69 | 0.95 (0.45) | 3.28 |
| Thiodicarb | Sf_Bra | 540 | 44.69 | 39.73 - 50.28 | 7.38 (0.93) |  |
|  | Sf_Des | 288 | 123.10 | 111.20 - 136.20 | 3.89 (0.54) | 2.75 |
| Spinosad | Sf_Bra | 396 | 5.51 | 4.64 - 6.53 | 2.36 (0.85) |  |
|  | Sf_Des | 396 | 10.16 | 8.87 - 11.63 | 5.18 (0.81) | 1.84 |
| Flubendiamide | Sf_Bra | 401 | 1.94 | 1.77 - 2.12 | 4.06 (0.39) |  |
|  | Sf_Des | 401 | 3.47 | 3.14 - 3.83 | 2.69 (0.30) | 1.79 |
| Abamectin | Sf_Bra | 288 | 72.06 | 62.61 - 82.94 | 2.63 (0.34) |  |
|  | Sf_Des | 252 | 102.30 | 89.86 - 116.50 | 4.32 (0.50) | 1.42 |
| Chlorfenapyr | Sf_Bra | 401 | 36.91 | 29.52 - 46.15 | 3.37 (1.35) |  |
|  | Sf_Des | 401 | 52.07 | 41.15 - 65.89 | 2.15 (0.38) | 1.41 |
| Tetraniliprole | Sf_Bra | 252 | 2.01 | 1.34 - 3.04 | 4.33 (1.50) |  |
|  | Sf_Des | 252 | 2.15 | 1.28 - 3.61 | 4.60 (1.78) | 1.06 |
| Indoxacarb | Sf_Bra | 252 | 4.65 | 4.44 - 4.86 | 4.38 (0.33) |  |
|  | Sf_Des | 252 | 4.68 | 4.21 - 5.19 | 5.90 (1.21) | 1.01 |
| Chlorantraniliprole | Sf_Bra | 252 | 0.82 | 0.58 - 1.15 | 2.75 (1.14) |  |
|  | Sf_Des | 252 | 0.38 | 0.33 - 0.43 | 3.20 (0.36) | 0.47 |
| ^a^ 95 % confidence interval; ^b^ Resistance ratio (EC_50_ of Sf_Des strain divided by EC_50_ of Sf_Bra). | | | | | | |

**Table S4**

|  | **All** | **Sf_Bra** | **Sf_Des** |
| --- | --- | --- | --- |
| Total trinity 'genes' | 118,013 | 59,502 | 74,448 |
| Total trinity transcripts | 209,969 | 112,964 | 157,228 |
|  | **Based on all 'transcripts'** | | |
| Contig N50 | 2,163 | 2,462 | 1,849 |
| Median contig length | 424 | 506 | 485 |
| Average contig | 993 | 1,180 | 992 |
| Total assembled bases | 208,581,896 | 133,268,072 | 155,966,890 |
| TransDecoder CDS | 200,590 |  |  |

**Table S5**

| **Sample** | **Sequences** | **Pseudoaligned** | **Estimated average fragment length** | **Pseudoaligned (%)** | **Minimum one read** | **Minimum one read (%)** |
| --- | --- | --- | --- | --- | --- | --- |
| Sf-Bra1 | 19655136 | 17653881 | 160 | 89.82 | 40855 | 0.71010185 |
| Sf-Bra2 | 19627680 | 17763461 | 150 | 90.50 | 41611 | 0.72324191 |
| Sf-Bra3 | 19435796 | 17559102 | 149 | 90.34 | 41356 | 0.71880975 |
| Sf-Bra4 | 21115281 | 19138106 | 151 | 90.64 | 40873 | 0.71041471 |
| Sf-Bra5 | 19937286 | 18014753 | 153 | 90.36 | 38733 | 0.67321931 |
| Sf-Des1 | 19649711 | 17518417 | 154 | 89.15 | 48975 | 0.85123579 |
| Sf-Des2 | 20379104 | 18267552 | 143 | 89.64 | 49189 | 0.85495533 |
| Sf-Des3 | 19228811 | 17085079 | 143 | 88.85 | 48532 | 0.843536 |
| Sf-Des4 | 19106471 | 16822902 | 152 | 88.05 | 46888 | 0.81496159 |
| Sf-Des5 | 21070255 | 18591915 | 155 | 88.24 | 48034 | 0.83488024 |

**Table S6**

| **Category** | **Term** | **Ontology^a^** | **P-value** | **Up-regulated in category** | **Genes in category** |
| --- | --- | --- | --- | --- | --- |
| GO:0055114 | oxidation-reduction process | BP | 2.83E-09 | 277 | 1053 |
| GO:0008152 | metabolic process | BP | 8.50E-04 | 141 | 618 |
| GO:0006629 | lipid metabolic process | BP | 7.00E-05 | 70 | 299 |
| GO:0009725 | response to hormone | BP | 3.31E-03 | 58 | 273 |
| GO:0044237 | cellular metabolic process | BP | 9.37E-04 | 49 | 223 |
| GO:0006259 | DNA metabolic process | BP | 1.44E-03 | 49 | 233 |
| GO:0015074 | DNA integration | BP | 8.87E-06 | 48 | 183 |
| GO:0040003 | chitin-based cuticle development | BP | 2.35E-08 | 43 | 108 |
| GO:1901564 | organonitrogen compound metabolic process | BP | 2.95E-03 | 43 | 177 |
| GO:0035220 | wing disc development | BP | 5.20E-05 | 39 | 130 |
| GO:0046680 | response to DDT | BP | 8.62E-08 | 38 | 76 |
| GO:0006030 | chitin metabolic process | BP | 5.73E-06 | 33 | 89 |
| GO:0035149 | lumen formation, open tracheal system | BP | 6.03E-06 | 33 | 79 |
| GO:0031000 | response to caffeine | BP | 3.08E-04 | 33 | 97 |
| GO:0006805 | xenobiotic metabolic process | BP | 1.10E-03 | 33 | 111 |
| GO:0071704 | organic substance metabolic process | BP | 5.92E-03 | 33 | 148 |
| GO:0007391 | dorsal closure | BP | 8.60E-03 | 28 | 104 |
| GO:0042572 | retinol metabolic process | BP | 3.39E-03 | 27 | 81 |
| GO:0008210 | estrogen metabolic process | BP | 1.94E-04 | 26 | 66 |
| GO:0044248 | cellular catabolic process | BP | 5.22E-03 | 26 | 107 |
| GO:0017143 | insecticide metabolic process | BP | 2.24E-05 | 24 | 54 |
| GO:0048252 | lauric acid metabolic process | BP | 1.74E-04 | 24 | 54 |
| GO:0030199 | collagen fibril organization | BP | 2.70E-03 | 21 | 59 |
| GO:0002118 | aggressive behavior | BP | 7.06E-03 | 21 | 75 |
| GO:0040040 | thermosensory behavior | BP | 8.46E-03 | 20 | 62 |
| GO:0042759 | long-chain fatty acid biosynthetic process | BP | 6.43E-04 | 19 | 50 |
| GO:0035002 | liquid clearance, open tracheal system | BP | 3.50E-03 | 19 | 52 |
| GO:0042573 | retinoic acid metabolic process | BP | 1.63E-03 | 18 | 44 |
| GO:0046949 | fatty-acyl-CoA biosynthetic process | BP | 9.56E-03 | 18 | 49 |
| GO:0015879 | carnitine transport | BP | 2.52E-04 | 17 | 39 |
| GO:0006706 | steroid catabolic process | BP | 1.05E-03 | 17 | 42 |
| GO:0035336 | long-chain fatty-acyl-CoA metabolic process | BP | 2.43E-03 | 17 | 45 |
| GO:0010025 | wax biosynthetic process | BP | 3.29E-03 | 17 | 47 |
| GO:0070989 | oxidative demethylation | BP | 3.33E-03 | 16 | 41 |
| GO:0030708 | germarium-derived female germ-line cyst encapsulation | BP | 1.23E-03 | 15 | 31 |
| GO:0042445 | hormone metabolic process | BP | 4.66E-03 | 14 | 48 |
| GO:1901362 | organic cyclic compound biosynthetic process | BP | 8.11E-04 | 13 | 33 |
| GO:0042738 | exogenous drug catabolic process | BP | 1.01E-03 | 13 | 28 |
| GO:0002933 | lipid hydroxylation | BP | 3.04E-03 | 13 | 30 |
| GO:0035204 | negative regulation of lamellocyte differentiation | BP | 5.62E-03 | 13 | 30 |
| GO:0019438 | aromatic compound biosynthetic process | BP | 1.39E-04 | 12 | 26 |
| GO:0072382 | minus-end-directed vesicle transport along microtubule | BP | 2.16E-03 | 12 | 21 |
| GO:0042074 | cell migration involved in gastrulation | BP | 4.91E-03 | 12 | 32 |
| GO:0007282 | cystoblast division | BP | 8.57E-03 | 12 | 25 |
| GO:0048803 | imaginal disc-derived male genitalia morphogenesis | BP | 8.74E-03 | 12 | 31 |
| GO:0007440 | foregut morphogenesis | BP | 9.89E-03 | 12 | 30 |

**Table S6_ cont.**

| **Category** | **Term** | **Ontology^a^** | **P-value** | **Up-regulated in category** | **Genes in category** |
| --- | --- | --- | --- | --- | --- |
| GO:0016098 | monoterpenoid metabolic process | BP | 5.07E-04 | 11 | 21 |
| GO:0006189 | 'de novo' IMP biosynthetic process | BP | 3.55E-03 | 11 | 21 |
| GO:0090235 | regulation of metaphase plate congression | BP | 4.30E-03 | 11 | 19 |
| GO:1990048 | anterograde neuronal dense core vesicle transport | BP | 5.50E-03 | 11 | 21 |
| GO:0070647 | protein modification by small protein conjugation or removal | BP | 6.28E-03 | 11 | 28 |
| GO:0051296 | establishment of meiotic spindle orientation | BP | 9.29E-03 | 11 | 23 |
| GO:0009822 | alkaloid catabolic process | BP | 9.56E-03 | 11 | 27 |
| GO:0070789 | metula development | BP | 3.95E-03 | 10 | 17 |
| GO:0098657 | import into cell | BP | 4.24E-03 | 10 | 32 |
| GO:0012501 | programmed cell death | BP | 8.34E-03 | 10 | 31 |
| GO:0051237 | maintenance of RNA location | BP | 9.95E-03 | 10 | 18 |
| GO:0000393 | spliceosomal conformational changes to generate catalytic conformation | BP | 6.89E-04 | 9 | 13 |
| GO:0021682 | nerve maturation | BP | 1.03E-03 | 9 | 13 |
| GO:0010838 | positive regulation of keratinocyte proliferation | BP | 2.10E-03 | 9 | 15 |
| GO:0009395 | phospholipid catabolic process | BP | 3.06E-03 | 9 | 18 |
| GO:1901563 | response to camptothecin | BP | 4.07E-03 | 9 | 17 |
| GO:0006145 | purine nucleobase catabolic process | BP | 6.79E-03 | 9 | 19 |
| GO:0002213 | defense response to insect | BP | 8.50E-03 | 9 | 18 |
| GO:0018130 | heterocycle biosynthetic process | BP | 5.77E-03 | 8 | 18 |
| GO:0042335 | cuticle development | BP | 6.36E-03 | 8 | 16 |
| GO:0021943 | formation of radial glial scaffolds | BP | 9.15E-03 | 8 | 16 |
| GO:0051608 | histamine transport | BP | 6.25E-03 | 7 | 15 |
| GO:0006029 | proteoglycan metabolic process | BP | 9.21E-03 | 7 | 12 |
| GO:0042435 | indole-containing compound biosynthetic process | BP | 1.74E-03 | 6 | 9 |
| GO:1905000 | regulation of membrane repolarization during atrial cardiac muscle cell action potential | BP | 1.77E-03 | 6 | 7 |
| GO:0018212 | peptidyl-tyrosine modification | BP | 1.96E-03 | 5 | 6 |
| GO:0042743 | hydrogen peroxide metabolic process | BP | 3.93E-03 | 5 | 10 |
| GO:0036150 | phosphatidylserine acyl-chain remodeling | BP | 6.38E-03 | 5 | 6 |
| GO:0043227 | membrane-bounded organelle | CC | 8.75E-04 | 95 | 484 |
| GO:0005903 | brush border | CC | 1.50E-03 | 58 | 213 |
| GO:0031012 | extracellular matrix | CC | 2.06E-06 | 56 | 174 |
| GO:0005588 | collagen type V trimer | CC | 9.92E-03 | 5 | 6 |
| GO:0005778 | peroxisomal membrane | CC | 1.61E-03 | 32 | 102 |
| GO:0000974 | Prp19 complex | CC | 1.53E-03 | 15 | 36 |
| GO:0036186 | early phagosome membrane | CC | 1.61E-03 | 12 | 21 |
| GO:0035182 | female germline ring canal outer rim | CC | 3.26E-03 | 12 | 23 |
| GO:0061474 | phagolysosome membrane | CC | 3.91E-03 | 12 | 24 |
| GO:0001411 | hyphal tip | CC | 4.42E-03 | 11 | 21 |
| GO:0032992 | protein-carbohydrate complex | CC | 8.19E-04 | 7 | 12 |
| GO:0005592 | collagen type XI trimer | CC | 2.20E-03 | 6 | 7 |
| GO:0016491 | oxidoreductase activity | MF | 1.77E-03 | 146 | 628 |
| GO:0020037 | heme binding | MF | 3.39E-08 | 107 | 341 |
| GO:0005506 | iron ion binding | MF | 3.36E-07 | 103 | 339 |
| GO:0042302 | structural constituent of cuticle | MF | 9.25E-32 | 93 | 159 |
| GO:0016705 | oxidoreductase activity, acting on paired donors, with incorporation or reduction of molecular oxygen | MF | 2.44E-08 | 86 | 242 |
| GO:0052689 | carboxylic ester hydrolase activity | MF | 3.66E-03 | 52 | 211 |

**Figure S6_cont.**

| **Category** | **Term** | **Ontology^a^** | **P-value** | **Up-regulated in category** | **Genes in category** |
| --- | --- | --- | --- | --- | --- |
| GO:0004518 | nuclease activity | MF | 2.72E-04 | 51 | 210 |
| GO:0005215 | transporter activity | MF | 8.39E-03 | 43 | 211 |
| GO:0008061 | chitin binding | MF | 2.78E-06 | 42 | 116 |
| GO:0008201 | heparin binding | MF | 9.71E-03 | 42 | 153 |
| GO:0004497 | monooxygenase activity | MF | 2.35E-04 | 39 | 125 |
| GO:0030246 | carbohydrate binding | MF | 6.27E-04 | 39 | 126 |
| GO:0008010 | structural constituent of chitin-based larval cuticle | MF | 5.78E-17 | 38 | 53 |
| GO:0008011 | structural constituent of pupal chitin-based cuticle | MF | 1.24E-16 | 35 | 47 |
| GO:0016758 | transferase activity, transferring hexosyl groups | MF | 2.45E-03 | 33 | 103 |
| GO:0018685 | alkane 1-monooxygenase activity | MF | 5.07E-05 | 27 | 61 |
| GO:0080019 | fatty-acyl-CoA reductase (alcohol-forming) activity | MF | 2.82E-04 | 23 | 56 |
| GO:0016627 | oxidoreductase activity, acting on the CH-CH group of donors | MF | 2.30E-03 | 22 | 57 |
| GO:0005496 | steroid binding | MF | 5.56E-04 | 19 | 47 |
| GO:0005504 | fatty acid binding | MF | 7.73E-03 | 19 | 51 |
| GO:0005214 | structural constituent of chitin-based cuticle | MF | 2.29E-08 | 18 | 28 |
| GO:0004177 | aminopeptidase activity | MF | 1.86E-03 | 18 | 51 |
| GO:0015651 | quaternary ammonium group transmembrane transporter activity | MF | 8.43E-06 | 16 | 32 |
| GO:0050062 | long-chain-fatty-acyl-CoA reductase activity | MF | 8.91E-04 | 16 | 38 |
| GO:0050649 | testosterone 6-beta-hydroxylase activity | MF | 2.16E-03 | 15 | 36 |
| GO:0015293 | symporter activity | MF | 7.48E-03 | 15 | 55 |
| GO:0101020 | estrogen 16-alpha-hydroxylase activity | MF | 6.58E-04 | 14 | 29 |
| GO:0000386 | second spliceosomal transesterification activity | MF | 1.06E-03 | 14 | 28 |
| GO:0008186 | RNA-dependent ATPase activity | MF | 1.98E-03 | 14 | 33 |
| GO:0008401 | retinoic acid 4-hydroxylase activity | MF | 1.06E-03 | 13 | 27 |
| GO:0030343 | vitamin D3 25-hydroxylase activity | MF | 1.95E-03 | 13 | 28 |
| GO:0070006 | metalloaminopeptidase activity | MF | 9.31E-03 | 13 | 34 |
| GO:0017070 | U6 snRNA binding | MF | 3.29E-04 | 12 | 19 |
| GO:0008395 | steroid hydroxylase activity | MF | 1.79E-03 | 12 | 32 |
| GO:0034875 | caffeine oxidase activity | MF | 2.23E-04 | 11 | 19 |
| GO:0030619 | U1 snRNA binding | MF | 5.19E-04 | 11 | 17 |
| GO:0101021 | estrogen 2-hydroxylase activity | MF | 2.92E-03 | 11 | 23 |
| GO:0070330 | aromatase activity | MF | 1.18E-03 | 10 | 17 |
| GO:0015103 | inorganic anion transmembrane transporter activity | MF | 2.04E-03 | 10 | 28 |
| GO:0070576 | vitamin D 24-hydroxylase activity | MF | 8.47E-03 | 10 | 26 |
| GO:0033791 | 3alpha,7alpha,12alpha-trihydroxy-5beta-cholestanoyl-CoA 24-hydroxylase activity | MF | 9.76E-03 | 10 | 19 |
| GO:0030623 | U5 snRNA binding | MF | 1.34E-03 | 9 | 13 |
| GO:0030620 | U2 snRNA binding | MF | 1.61E-03 | 9 | 14 |
| GO:0004558 | alpha-1,4-glucosidase activity | MF | 4.92E-03 | 8 | 16 |
| GO:0030023 | extracellular matrix constituent conferring elasticity | MF | 9.42E-03 | 8 | 17 |
| GO:0004167 | dopachrome isomerase activity | MF | 2.05E-03 | 7 | 12 |
| GO:0004641 | phosphoribosylformylglycinamidine cyclo-ligase activity | MF | 5.20E-03 | 6 | 8 |
| GO:0004644 | phosphoribosylglycinamide formyltransferase activity | MF | 5.20E-03 | 6 | 8 |
| GO:1904399 | heparan sulfate binding | MF | 2.23E-03 | 5 | 5 |
| GO:0034988 | Fc-gamma receptor I complex binding | MF | 5.41E-03 | 5 | 6 |
| GO:0003823 | antigen binding | MF | 9.89E-03 | 5 | 7 |

**Table S7**

| **Category** | **Term** | **Ontology** | **P-value** | **Down-regulated in category** | **Genes in category** |
| --- | --- | --- | --- | --- | --- |
| GO:0055114 | oxidation-reduction process | BP | 6.55E-05 | 113 | 1053 |
| GO:0008152 | metabolic process | BP | 2.12E-05 | 72 | 618 |
| GO:0006508 | proteolysis | BP | 4.77E-03 | 65 | 666 |
| GO:0015074 | DNA integration | BP | 1.36E-14 | 42 | 183 |
| GO:0005975 | carbohydrate metabolic process | BP | 9.42E-05 | 40 | 292 |
| GO:0006259 | DNA metabolic process | BP | 1.45E-07 | 36 | 233 |
| GO:0042742 | defense response to bacterium | BP | 4.99E-03 | 30 | 272 |
| GO:0007584 | response to nutrient | BP | 3.91E-04 | 29 | 199 |
| GO:0032196 | transposition | BP | 3.96E-11 | 28 | 113 |
| GO:0009617 | response to bacterium | BP | 1.84E-03 | 27 | 217 |
| GO:0046686 | response to cadmium ion | BP | 4.15E-04 | 26 | 180 |
| GO:0043627 | response to estrogen | BP | 1.45E-04 | 25 | 162 |
| GO:0044249 | cellular biosynthetic process | BP | 5.57E-03 | 23 | 197 |
| GO:0008202 | steroid metabolic process | BP | 1.08E-04 | 20 | 120 |
| GO:0044248 | cellular catabolic process | BP | 9.35E-04 | 16 | 107 |
| GO:0006633 | fatty acid biosynthetic process | BP | 3.83E-03 | 16 | 100 |
| GO:0017085 | response to insecticide | BP | 1.25E-04 | 14 | 66 |
| GO:0042572 | retinol metabolic process | BP | 3.55E-03 | 14 | 81 |
| GO:0009744 | response to sucrose | BP | 4.23E-03 | 13 | 79 |
| GO:0008610 | lipid biosynthetic process | BP | 5.27E-03 | 13 | 87 |
| GO:1901575 | organic substance catabolic process | BP | 5.94E-03 | 13 | 94 |
| GO:0010288 | response to lead ion | BP | 9.87E-03 | 12 | 81 |
| GO:0010040 | response to iron(II) ion | BP | 2.47E-03 | 11 | 53 |
| GO:0071385 | cellular response to glucocorticoid stimulus | BP | 1.17E-03 | 10 | 47 |
| GO:0017143 | insecticide metabolic process | BP | 6.92E-03 | 10 | 54 |
| GO:0031288 | sorocarp morphogenesis | BP | 8.50E-03 | 10 | 55 |
| GO:0042574 | retinal metabolic process | BP | 8.96E-03 | 10 | 56 |
| GO:0007021 | tubulin complex assembly | BP | 3.42E-05 | 9 | 24 |
| GO:0006066 | alcohol metabolic process | BP | 5.51E-05 | 9 | 27 |
| GO:0001507 | acetylcholine catabolic process in synaptic cleft | BP | 1.10E-03 | 9 | 32 |
| GO:0090304 | nucleic acid metabolic process | BP | 4.55E-03 | 9 | 52 |
| GO:0009410 | response to xenobiotic stimulus | BP | 5.21E-03 | 9 | 59 |
| GO:0006707 | cholesterol catabolic process | BP | 6.14E-03 | 9 | 42 |
| GO:0042573 | retinoic acid metabolic process | BP | 6.70E-03 | 9 | 44 |
| GO:0010045 | response to nickel cation | BP | 7.90E-03 | 9 | 46 |
| GO:0052695 | cellular glucuronidation | BP | 6.47E-03 | 8 | 40 |
| GO:0044241 | lipid digestion | BP | 7.00E-03 | 8 | 41 |
| GO:0044245 | polysaccharide digestion | BP | 8.69E-03 | 8 | 34 |
| GO:0071294 | cellular response to zinc ion | BP | 1.82E-03 | 7 | 31 |
| GO:0009809 | lignin biosynthetic process | BP | 2.06E-03 | 7 | 22 |
| GO:0034754 | cellular hormone metabolic process | BP | 9.83E-03 | 7 | 38 |
| GO:0007023 | post-chaperonin tubulin folding pathway | BP | 1.17E-05 | 6 | 8 |
| GO:0071378 | cellular response to growth hormone stimulus | BP | 3.71E-03 | 6 | 20 |
| GO:0030422 | production of siRNA involved in RNA interference | BP | 4.03E-03 | 6 | 20 |
| GO:0070980 | biphenyl catabolic process | BP | 4.07E-03 | 6 | 21 |
| GO:0045071 | negative regulation of viral genome replication | BP | 4.33E-03 | 6 | 21 |
| GO:0007304 | chorion-containing eggshell formation | BP | 4.56E-03 | 6 | 23 |
| GO:0071236 | cellular response to antibiotic | BP | 5.08E-03 | 6 | 25 |
| GO:0042760 | very long-chain fatty acid catabolic process | BP | 6.29E-03 | 6 | 20 |
| GO:0006069 | ethanol oxidation | BP | 9.04E-03 | 6 | 22 |

**Table S7_cont.**

| **Category** | **Term** | **Ontology** | **P-value** | **Down-regulated in category** | **Genes in category** |
| --- | --- | --- | --- | --- | --- |
| GO:0051901 | positive regulation of mitochondrial depolarization | BP | 9.06E-05 | 5 | 9 |
| GO:0036267 | invasive filamentous growth | BP | 2.67E-03 | 5 | 12 |
| GO:0097054 | L-glutamate biosynthetic process | BP | 2.67E-03 | 5 | 12 |
| GO:0048194 | Golgi vesicle budding | BP | 2.77E-03 | 5 | 14 |
| GO:0006751 | glutathione catabolic process | BP | 3.76E-03 | 5 | 14 |
| GO:0032223 | negative regulation of synaptic transmission, cholinergic | BP | 5.01E-03 | 5 | 16 |
| GO:0002175 | protein localization to paranode region of axon | BP | 5.02E-03 | 5 | 17 |
| GO:0030245 | cellulose catabolic process | BP | 6.44E-03 | 5 | 16 |
| GO:0000467 | exonucleolytic trimming to generate mature 3'-end of 5.8S rRNA from tricistronic rRNA transcript (SSU-rRNA, 5.8S rRNA, LSU-rRNA) | BP | 7.91E-03 | 5 | 17 |
| GO:0019676 | ammonia assimilation cycle | BP | 9.45E-03 | 5 | 16 |
| GO:0043030 | regulation of macrophage activation | BP | 9.89E-03 | 5 | 19 |
| GO:0005777 | peroxisome | CC | 1.07E-03 | 28 | 215 |
| GO:0070578 | RISC-loading complex | CC | 2.03E-03 | 6 | 17 |
| GO:0042600 | chorion | CC | 5.97E-03 | 5 | 16 |
| GO:0005615 | extracellular space | CC | 7.14E-03 | 89 | 969 |
| GO:0005604 | basement membrane | CC | 9.06E-03 | 16 | 112 |
| GO:0016740 | transferase activity | MF | 6.43E-09 | 69 | 542 |
| GO:0004518 | nuclease activity | MF | 3.81E-06 | 32 | 210 |
| GO:0004497 | monooxygenase activity | MF | 8.02E-06 | 24 | 125 |
| GO:0004553 | hydrolase activity, hydrolyzing O-glycosyl compounds | MF | 4.28E-05 | 25 | 135 |
| GO:0016787 | hydrolase activity | MF | 9.33E-05 | 61 | 570 |
| GO:0001972 | retinoic acid binding | MF | 1.43E-04 | 12 | 43 |
| GO:0004252 | serine-type endopeptidase activity | MF | 3.07E-04 | 37 | 279 |
| GO:0016779 | nucleotidyltransferase activity | MF | 3.08E-04 | 23 | 157 |
| GO:0102799 | glucosinolate glucohydrolase activity | MF | 5.54E-04 | 5 | 9 |
| GO:0008395 | steroid hydroxylase activity | MF | 7.85E-04 | 8 | 32 |
| GO:0047782 | coniferin beta-glucosidase activity | MF | 9.49E-04 | 7 | 19 |
| GO:0017168 | 5-oxoprolinase (ATP-hydrolyzing) activity | MF | 1.19E-03 | 5 | 10 |
| GO:0015925 | galactosidase activity | MF | 1.25E-03 | 5 | 11 |
| GO:0052689 | carboxylic ester hydrolase activity | MF | 1.66E-03 | 27 | 211 |
| GO:0000016 | lactase activity | MF | 1.73E-03 | 10 | 42 |
| GO:0005488 | binding | MF | 1.78E-03 | 61 | 712 |
| GO:0140097 | catalytic activity, acting on DNA | MF | 2.21E-03 | 7 | 33 |
| GO:0019137 | thioglucosidase activity | MF | 2.27E-03 | 5 | 12 |
| GO:0016758 | transferase activity, transferring hexosyl groups | MF | 2.29E-03 | 17 | 103 |
| GO:0017171 | serine hydrolase activity | MF | 2.38E-03 | 7 | 26 |
| GO:0015923 | mannosidase activity | MF | 2.54E-03 | 7 | 24 |
| GO:0003824 | catalytic activity | MF | 2.60E-03 | 68 | 699 |
| GO:0004806 | triglyceride lipase activity | MF | 2.68E-03 | 16 | 92 |
| GO:0016705 | oxidoreductase activity, acting on paired donors, with incorporation or reduction of molecular oxygen | MF | 2.78E-03 | 32 | 242 |
| GO:0015020 | glucuronosyltransferase activity | MF | 3.23E-03 | 10 | 46 |
| GO:0005518 | collagen binding | MF | 3.38E-03 | 13 | 78 |

| **Category** | **Term** | **Ontology** | **P-value** | **Down-regulated in category** | **Genes in category** |
| --- | --- | --- | --- | --- | --- |
| GO:0042626 | ATPase activity, coupled to transmembrane movement of substances | MF | 3.73E-03 | 12 | 75 |
| GO:0043878 | glyceraldehyde-3-phosphate dehydrogenase (NAD+) (non-phosphorylating) activity | MF | 3.84E-03 | 5 | 12 |
| GO:0008390 | testosterone 16-alpha-hydroxylase activity | MF | 4.14E-03 | 5 | 16 |
| GO:0030343 | vitamin D3 25-hydroxylase activity | MF | 5.27E-03 | 7 | 28 |
| GO:0003990 | acetylcholinesterase activity | MF | 5.51E-03 | 9 | 41 |
| GO:0005506 | iron ion binding | MF | 5.67E-03 | 39 | 339 |
| GO:0020037 | heme binding | MF | 5.77E-03 | 39 | 341 |
| GO:0004190 | aspartic-type endopeptidase activity | MF | 5.77E-03 | 8 | 44 |
| GO:0015928 | fucosidase activity | MF | 6.03E-03 | 5 | 15 |
| GO:0016162 | cellulose 1,4-beta-cellobiosidase activity | MF | 6.03E-03 | 5 | 15 |
| GO:0097599 | xylanase activity | MF | 6.03E-03 | 5 | 15 |
| GO:0016616 | oxidoreductase activity, acting on the CH-OH group of donors, NAD or NADP as acceptor | MF | 6.13E-03 | 14 | 93 |
| GO:0016040 | glutamate synthase (NADH) activity | MF | 6.30E-03 | 5 | 14 |
| GO:0016491 | oxidoreductase activity | MF | 6.52E-03 | 63 | 628 |
| GO:0035197 | siRNA binding | MF | 6.68E-03 | 5 | 16 |
| GO:0004028 | 3-chloroallyl aldehyde dehydrogenase activity | MF | 9.24E-03 | 6 | 22 |
| GO:0008061 | chitin binding | MF | 9.30E-03 | 16 | 116 |
| GO:0048487 | beta-tubulin binding | MF | 9.53E-03 | 10 | 58 |
| GO:0032451 | demethylase activity | MF | 9.63E-03 | 5 | 19 |

**Table S7_cont.**

**Figure S1**


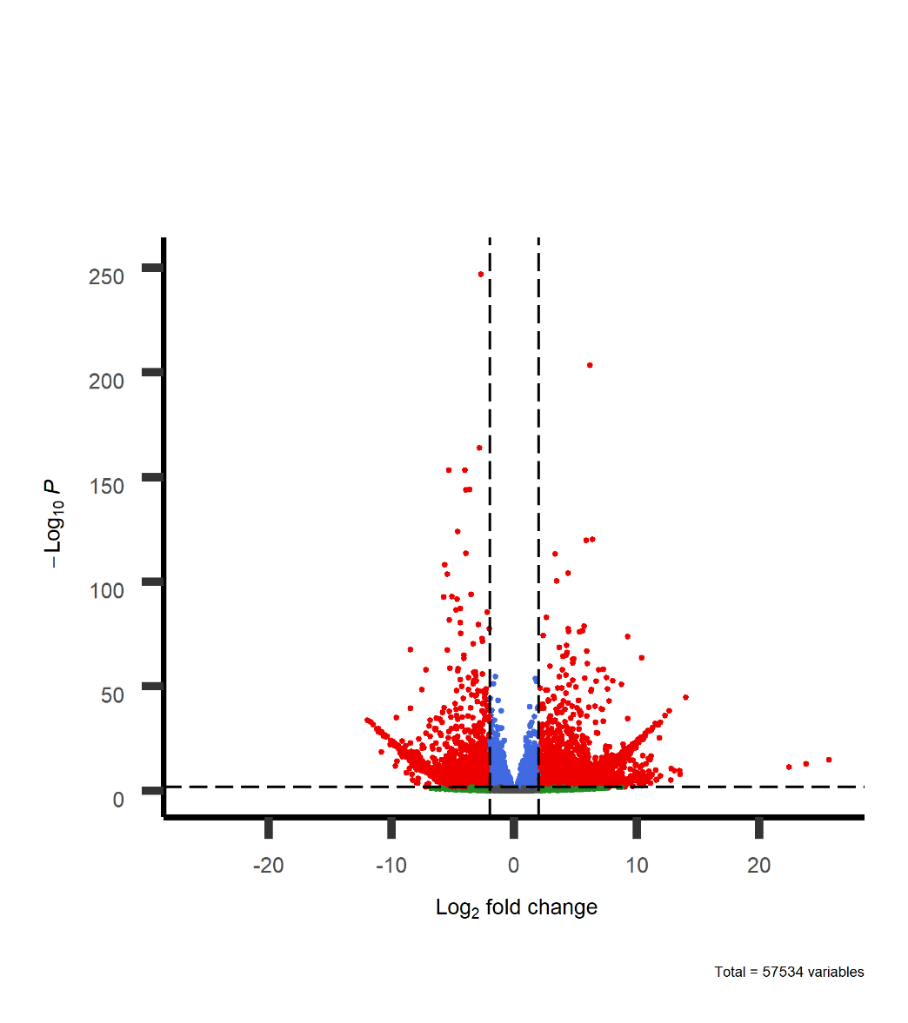


**Figure S2**


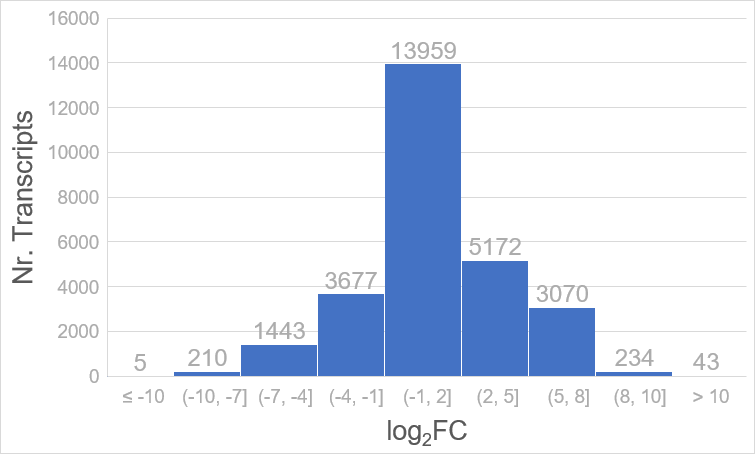


**Figure S3**

**CYP9-like**

**
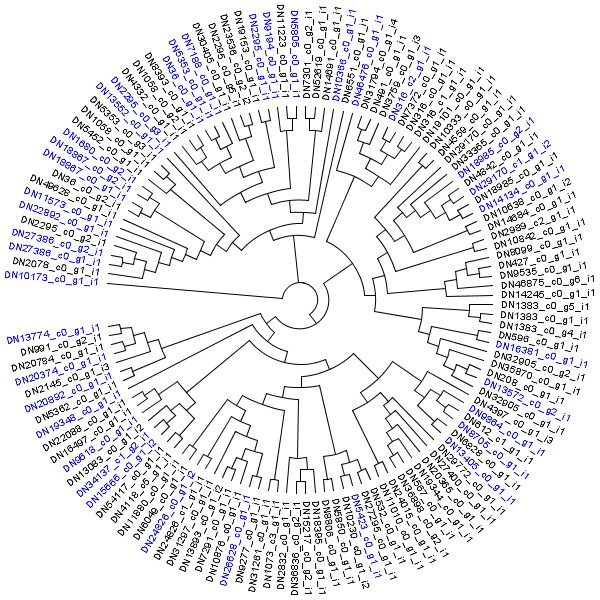
**
